# Supplementary material for: MEF2 Is an In Vivo Immune-Metabolic Switch
Source: Cell. 2013 Oct 10;155(2):435–47. doi: 10.1016/j.cell.2013.09.007 (PMC3807682; doi:10.1016/j.cell.2013.09.007)
Supplement: Document S1. Article plus Supplemental Information [file mmc7.pdf]

MEF2 Is an In Vivo Immune-Metabolic Switch

Rebecca I. Clark,[1,4] Sharon W.S. Tan,[1,6] Claire B. Péan,[1] Urmas Roostalu,[1,7] Valérie Vivancos,[1] Kévin Bronda,[1,8] Martina Pilátová,[1,9] Jingqi Fu,[2] David W. Walker,[4,5] Rebecca Berdeaux,[2,3] Frédéric Geissmann,[1] and Marc S. Dionne[1,*]
[1]Centre for the Molecular and Cellular Biology of Inflammation and Peter Gorer Department of Immunobiology, King's College London School of Medicine, London SE1 1UL, UK
[2]Department of Integrative Biology and Pharmacology
[3]Graduate School of Biomedical Sciences
University of Texas Health Science Center at Houston, Houston, TX 77030, USA
[4]Department of Integrative Biology and Physiology
[5]Molecular Biology Institute
University of California, Los Angeles, Los Angeles, CA 90095, USA
[6]Present address: Program in Emerging Infectious Diseases, Duke-NUS Graduate Medical School, Singapore 169857, Singapore
[7]Present address: Department of Cell and Developmental Biology, University College London, London WC1E 6BT, UK
[8]Present address: IMI Programme, UFR de Chimie et de Biologie, Université Joseph Fourier, 38 041 Grenoble Cedex 9, France
[9]Present address: Randall Division of Molecular Biophysics, King's College London, London SE1 1UL, UK
*Correspondence: marc.dionne@kcl.ac.uk
http://dx.doi.org/10.1016/j.cell.2013.09.007
This is an open-access article distributed under the terms of the Creative Commons Attribution License, which permits unrestricted use, distribution, and reproduction in any medium, provided the original author and source are credited.

SUMMARY

Infections disturb metabolic homeostasis in many contexts, but the underlying connections are not completely understood. To address this, we use paired genetic and computational screens in *Drosophila* to identify transcriptional regulators of immunity and pathology and their associated target genes and physiologies. We show that *Mef2* is required in the fat body for anabolic function and the immune response. Using genetic and biochemical approaches, we find that MEF2 is phosphorylated at a conserved site in healthy flies and promotes expression of lipogenic and glycogenic enzymes. Upon infection, this phosphorylation is lost, and the activity of MEF2 changes—MEF2 now associates with the TATA binding protein to bind a distinct TATA box sequence and promote antimicrobial peptide expression. The loss of phosphorylated MEF2 contributes to loss of anabolic enzyme expression in Gram-negative bacterial infection. MEF2 is thus a critical transcriptional switch in the adult fat body between metabolism and immunity.

INTRODUCTION

Metabolic regulation is tightly and ubiquitously linked with immune responses and inflammatory signaling (Hotamisligil, 2006). Prolonged or excessive immune activation can drive metabolic disruption and cause wasting of fatty and lean tissues. This effect is seen in many human infections; it is particularly prominent in Gram-negative sepsis and in persistent bacterial infections such as tuberculosis (Schwenk and Macallan, 2000; Tappy and Chioléro, 2007). Because of the many etiologies behind infection-induced cachexia, numerous molecular mechanisms have been proposed to underlie this condition, with signals including lipid mediators and cytokines and transcription factors including FOXO, NF-κB, AP-1, Stats, and nuclear receptors acting singly or in combination (Tracey and Cerami, 1994; Vallerie and Hotamisligil, 2010; Van den Berghe, 2002). It has been a major challenge to the field to experimentally link any one of these molecular mechanisms to observed metabolic dysfunction following infection in vivo.

Chronic or acute infection disrupts systemic metabolism in *Drosophila* as well as in vertebrates. We have previously shown that infection with *Mycobacterium marinum* causes metabolic disruptions in *Drosophila* resulting, in part, from a systemic loss of AKT activity (Dionne et al., 2006). This promotes pathological FOXO activation and an inability to produce new metabolic stores. Activation of the *Toll* pathway in the fly fat body, either genetically or by infection, is sufficient to partially phenocopy this effect, both at the level of AKT activity and metabolic storage (DiAngelo et al., 2009). *Listeria* infection causes similar metabolic pathology (Chambers et al., 2012). The function of the link between immune activation and loss of anabolic signaling activity is unclear, especially because FOXO is able to activate antimicrobial peptide expression but is not required for resistance to infection (Becker et al., 2010). How other anabolic or catabolic pathways are altered by infection in flies is also unknown, though it is clear that there are other common regulators of these processes, at least at the level of the whole organism (Rynes et al., 2012).

We used a paired screening strategy to identify the pathways and transcriptional networks that regulate host physiology in vivo in response to infection. One screen used computational analysis of gene expression after different immune challenges to identify coregulated genes and their predicted transcriptional

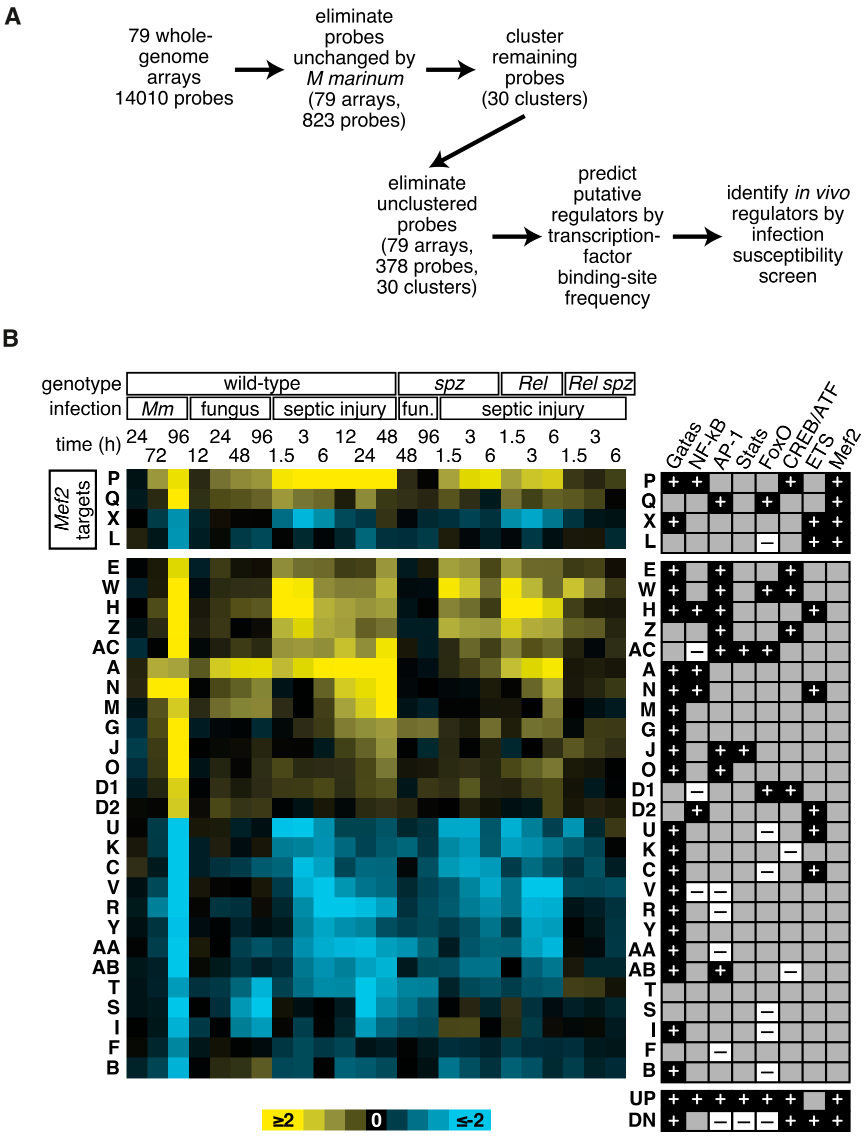

Figure 1. Computational Predictions for Transcriptional Regulators of Infection

(A) Diagram of our approach.

(B) Expression and predicted regulation of gene clusters. Left, $\log_2$(infection/control) of the median of each cluster in each infection condition (yellow indicates increased mRNA level after infection, blue indicates decreased). Right, predicted transcription-factor associations of each cluster, as well as predictions derived from analysis of the entire sets of transcripts up- and downregulated after *M. marinum* infection. + indicates $p \leq 0.02$ (overrepresented sites), – indicates $0.98 \leq p$ (underrepresented sites). Genotypes: *spz*, *spätzle* (*Toll* pathway) mutant; *Rel*, *Relish* (*imd* pathway) mutant. Infections: *Mm*, *M.marinum*; fungus/fun., *Beauvaria bassiana*; septic injury, mixed *M. luteus* and *E. coli*. See also Figure S1 and Tables S1 and S2.

regulators. The other screen involved testing mycobacterial infection susceptibility of flies carrying targeted RNAi knockdowns of transcription factors and signaling intermediates. These screens converged on MEF2, a pleiotropic transcription factor originally characterized as a key factor in muscle development (Bour et al., 1995; Lilly et al., 1995; Molkentin et al., 1995). Our data reveal that MEF2 regulates immune and metabolic activities, as depletion of MEF2 in the fat body causes dramatic failures of systemic anabolism and immune function. This results from reduced expression of key metabolic enzymes and antimicrobial peptides, respectively. The choice between immune and metabolic target genes is dictated by phosphorylation of MEF2 at a conserved site. In healthy animals, MEF2 is phosphorylated at T20 and promotes expression of its metabolic targets, whereas infection results in T20 dephosphorylation and association with the TATA-binding protein (TBP) at a distinct TATA sequence of immune targets.

RESULTS

Computational and Functional Screens Reveal *Mef2* as a Regulator of Infection Susceptibility

To identify new factors in the response to infection, we performed two screens. In the first, we analyzed a large microarray data set reflecting whole-fly gene expression at multiple times after several different infections (for details, see Figure 1 and supporting material) (De Gregorio et al., 2001, 2002; Dionne et al., 2006). This data set included wild-type animals as well as loss-of-function mutants in the *imd* and *Toll* pathways, the two primary microbe-detection systems in the fly. These two pathways respond to different microbes and share no molecular components but regulate overlapping sets of target genes because each culminates in activation of a distinct NF-κB family member (Lemaitre and Hoffmann, 2007). We used fuzzy c-means clustering to find clusters of genes coregulated across biological conditions and then identified transcription factor binding sites overrepresented in the vicinity of the genes within each cluster (Figure 1 and Tables S1 and S2 available online). These binding sites represent potential regulators of the associated genes.

This type of computational analysis affords direct identification of transcriptional effectors, their targets, and regulated physiologies simultaneously without prior knowledge. This is reflected in the fact that many of our coregulated groups of genes correspond to clear biological functions. In keeping with our previous observation of metabolic dysregulation in *Mycobacterium marinum*-infected flies, metabolic functions were overrepresented in 6 of the 30 clusters (Figure S1), suggesting that the shared transcriptional regulators of these clusters may have important metabolic roles.

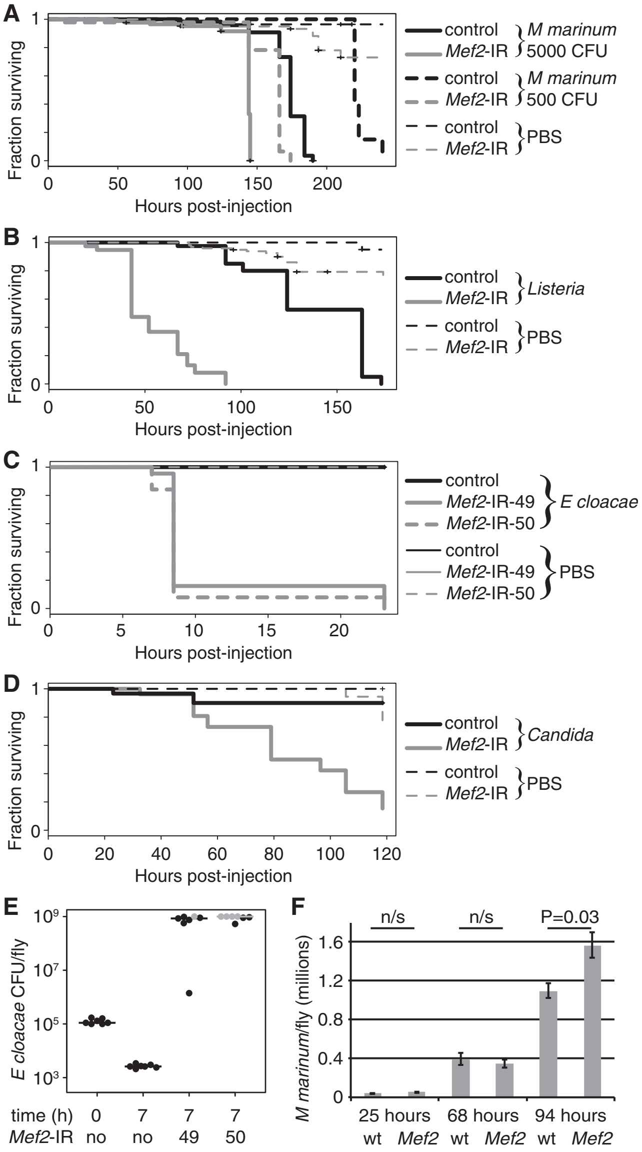

Figure 2. Infection Susceptibility of Fat Body *Mef2* Knockdowns

(A–D) Survival of *Mef2* knockdown (w^{1118}/Y ; c564/+ ; UAS-*Mef2*-IR/+) or control (w^{1118}/Y ; c564/+) flies after different infections. Two *Mef2* knockdown lines are shown for *E. cloacae*; all phenotypes were observed with both lines, but only one line is typically shown for simplicity. p values for differences in survival times between control and *Mef2* knockdowns by log-rank test: *M. marinum* 5,000 colony-forming units (CFU), $p = 3.89 \times 10^{-9}$; *M. marinum* 500 CFU, $p = 9.35 \times 10^{-14}$; *Listeria*, p = 0; *E. cloacae*, 15,549, p = 0; *E. cloacae*, 15,550, p = 0; *Candida*, $p = 5.07 \times 10^{-8}$.

(E) *E. cloacae* CFU per fly, input, and 7 hr after infection in driver-only controls and two *Mef2* knockdowns. Seven individual animals are shown for each condition. Line indicates the median. Points or lines in gray exceeded the maximum range of the assay (10^9 CFU/fly).

(F) *M. marinum* numbers per fly assayed by qRT-PCR after an initial infection with 5,000 CFU. Values are mean ± SEM.

See also Figure S2.

To test in vivo function of predicted regulators, we performed a secondary functional screen, testing time to death after *M. marinum* infection in flies with ubiquitous or fat-body-specific RNAi knockdown of 29 transcription factors or signals associated with specific factors predicted to directly regulate gene clusters after infection. Five genes gave clear survival phenotypes; of these, *Mef2* was particularly intriguing, being associated both with immune-response genes (cluster P) and with metabolic activities (cluster X). We further analyzed *Mef2* as a potential nodal point between immune activation and metabolic disruption.

Mef2 Regulates Susceptibility to Infection

The fat body, a homolog of mammalian liver and adipose tissue, is both the primary source of the inducible humoral immune response and the site of metabolic stores in the fly. Because strong loss-of-function mutants in *Mef2* are embryonic lethal (Bour et al., 1995; Lilly et al., 1995), we tested animals with *Mef2* RNAi knockdown driven by c564, a driver strongly expressed in the fat body (Hrdlicka et al., 2002). Flies carrying fat body *Mef2* RNAi exhibited a significant reduction in survival time after a high-dose *M. marinum* infection compared with driver-only controls (Figure 2A). This survival effect was strengthened at lower bacterial doses.

To determine whether MEF2 is generally required for survival after infection, we assayed survival of fat body *Mef2* knockdowns after other infections. In addition to *M. marinum*, these animals were compromised in response to a second intracellular pathogen, *Listeria monocytogenes* (Figure 2B). Moreover, unlike wild-type animals, they were killed by infection with either *Enterobacter cloacae* or *Candida albicans* (Figures 2C and 2D). Defense against these infections is mediated specifically by the *imd* and *Toll* pathways, respectively (Gottar et al., 2006). Sensitivity of flies lacking fat body *Mef2* to *E. cloacae*, in particular, was similar to that of *imd* pathway mutants (Figure S2A) (Gottar et al., 2006; Hedengren et al., 1999). This could be explained if these flies lacked the fat body, but we found no visible reduction in fat body mass or decrease in c564-driven GFP expression (Figure S2B). All phenotypes were tested with a second *Mef2* knockdown line, which gave similar or identical results in all cases (Figure 2C and data not shown). Both lines eliminated detectable fat body MEF2 protein when driven with c564 (Figure S2C). Other fat body drivers recapitulated the susceptibility to *E. cloacae* (Figure S2D), and the susceptibility resulting from *Mef2* knockdown was rescued by coexpression of wild-type *Mef2* (Figure S2E).

We then assayed bacterial numbers in animals infected with *E. cloacae* or *M. marinum*. In each case, more bacteria were present in *Mef2* knockdown animals (Figures 2E and 2F). The dramatic increase in *E. cloacae* number suggests that the effect on this infection may be fully accounted for by defects in bactericidal activity. By contrast, the modest difference observed in *M. marinum* number suggests that, in a more complex, chronic infection, MEF2 is also important in tolerance of pathology.

Mef2 Is Required for Normal Fat and Glycogen Storage

As mentioned earlier, our data set included many genes encoding metabolic activities. Cluster X, a predicted MEF2 target

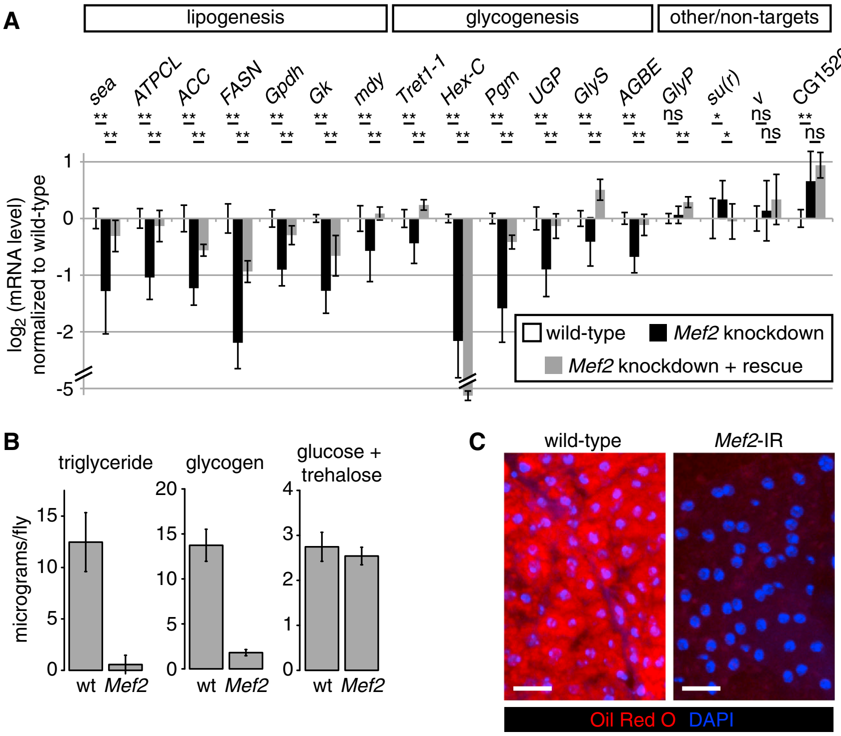

Figure 3. Metabolic Effects of *Mef2* Knockdown

(A) Expression of genes encoding enzymes of triglyceride and fatty acid synthesis, glucose uptake, and glycogen synthesis in control (w^{1118}/Y ; c564/+), *Mef2* knockdown (w^{1118}/Y ; c564/+ ; UAS-*Mef2*-IR/+), and rescued (w^{1118}/Y ; c564/ UAS-*Mef2.HA* ; UAS-*Mef2*-IR/+) flies, assayed by qRT-PCR. mRNA levels are normalized to the control genotype (w^{1118}/Y ; c564 / +) and expressed as $\log_2$. Values are mean ± SD. Significance levels: *$p < 0.01$ and **$p < 0.001$ by heteroscedastic unpaired two-tailed t test.

(B) Total triglyceride, glycogen, and free glucose measured by colorimetric enzymatic assay in *Mef2* knockdown flies compared with driver-only controls.

(C) Neutral lipids stained by oil red O in driver-only control and *Mef2* knockdown fat body. Red, oil red O; blue, DAPI. Scale bars, 20 μm.

See also Figure S3 and Table S3.

cluster, contained several key metabolic enzymes, including acetyl-CoA carboxylase (*ACC*), diacylglycerol acyltransferase (*mdy*), and fat body hexose kinase (*Hex-C*). In addition to being repressed by *M. marinum* infection, cluster X was rapidly and transiently downregulated 3–6 hr after septic injury (infection with a mixture of nonpathogenic Gram-positive and Gram-negative bacteria), with most genes returning to baseline within 24 hr (Figure 1B). In addition to genes in cluster X, genes encoding key anabolic enzymes in clusters Y and AA, as well as unclustered genes, were repressed after either *M. marinum* infection or septic injury. These observations led us to examine the effects of MEF2 on the expression of cluster X genes—and other anabolic enzymes showing similar expression—in the absence of infection.

Mef2 knockdown reduced expression of numerous key enzymes in fatty acid and triglyceride synthesis, including *ACC*, *mdy*, fatty acid synthase (*FASN*/CG3523), ATP citrate lyase (*ATPCL*), glycerol-3-phosphate dehydrogenase (*Gpdh*), glycerol kinase (*Gk*), and the mitochondrial citrate transporter (*sea*) (Figure 3A). A similar pattern was apparent for the enzymes of fat body glucose uptake and glycogen synthesis, including *Hex-C*, *Tret1-1* (one of two GLUT family members highly expressed in fat body [Chintapalli et al., 2007]), phosphoglucose mutase (*Pgm*), UDP-glucose pyrophosphorylase (*UGP*), glycogen synthase (*GlyS*/CG6904), and the glycogen branching enzyme (*AGBE*) (Figure 3A, black bars). The expression of almost all these genes was rescued by coexpression of wild-type *Mef2* (Figure 3A, gray bars); for a few targets, this rescue was partial, likely because the RNAi targeted the rescue transgene as well as the endogenous locus. Genes that were incompletely rescued or that showed paradoxical repression in the rescue-containing animals (*FASN* and *Hex-C*) could also be repressed by inducible expression of a *Mef2*-engrailed repressor fusion (Blanchard et al., 2010), confirming their dependence on *Mef2* for expression (Figure S3A). However, the fat body was intact and continued to express glycogen phosphorylase (GlyP), GFP driven by c564, and other fat-body-enriched genes, indicating that Mef2 is not required for development, survival, or overall identity of this tissue (Figures 3A and S2).

In keeping with reduced expression of enzymes of glycogenesis (*Hex-C*, *Pgm*, *UGP*, *GlyS*, and *AGBE*) and lipogenesis (*sea*, *ATPCL*, *ACC*, *FASN*, *Gpdh*, *Gk*, and *mdy*), unchallenged *Mef2* knockdown flies were almost entirely devoid of triglyceride and glycogen (Figures 3B, 3C, S3B, and S3C). These animals contained normal levels of free glucose and trehalose, suggesting that, even without glycogen or triglyceride stores, they can regulate circulating sugar (Figure 3B). These metabolic phenotypes could be seen, though more weakly, with a second fat body driver (Figures S3D and S3E; data not shown). Other fat body drivers may give weaker phenotypes because they knock down *Mef2* less efficiently or because c564 also knocks down *Mef2* in other tissues (for example, the gut, which is lipogenic and parts of which also exhibit c564-driven expression). As would be expected, *Mef2* knockdown animals died more rapidly than wild-type flies when starved (Figure S3F). However, they were not generally stress sensitive, exhibiting normal survival under either hyperoxia or heat stress (Figures S3G–S3J).

These data suggest that the statistical association of predicted *Mef2* sites with genes encoding key metabolic activities reflects a requirement for *Mef2* in normal expression of these genes and thus in normal metabolic function. Although MEF2 may regulate its metabolic targets indirectly, we favor direct regulation, as 12/13 of the identified metabolic target genes have high-quality MEF2 binding sites within their 5′ or 3′ flanking

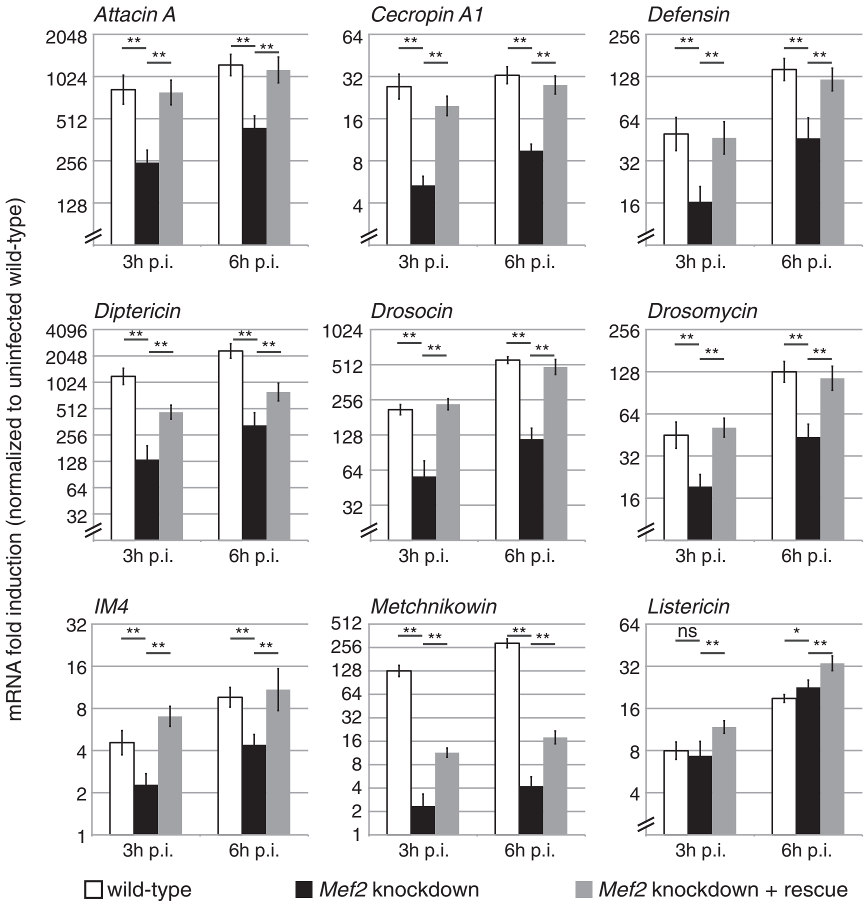

Figure 4. Effects of *Mef2* Knockdown on Infection-Induced Gene Expression

Expression of antimicrobial peptides in control, *Mef2* knockdown, and rescued flies 3 or 6 hr after injection of mixed *M. luteus* and *E. coli* assayed by qRT-PCR and normalized to uninjected controls (*Drosophila* genotypes as in Figure 3A). Values are mean ± SD. Significance levels: *p < 0.01 and **p < 0.001 by heteroscedastic unpaired two-tailed t test.

See also Figure S4.

regions or in introns (Table S3). The severe metabolic defect in *Mef2* knockdown animals likely contributes to their short lifespan after mycobacterial infection by decreasing their ability to withstand infection-induced wasting.

Mef2 Knockdown Impairs Humoral Immune Responses

Although *Mef2* has not previously been described as an immune regulator in *Drosophila*, our computational analysis also associated *Mef2* sites with cluster P, which contains many genes known to be targeted by NF-κB-like factors upon activation of the *imd* or *Toll* pathways, including the antimicrobial peptides *Defensin*, *Metchnikowin*, *Drosocin*, and *Attacin A.* These genes are typically strongly induced within 3 hr of septic injury.

To test whether MEF2 regulates these genes, we examined antimicrobial peptide response to septic injury in fat body *Mef2* knockdown flies. We again infected flies with a mixture of the *imd* agonist *Escherichia coli* and the *Toll* agonist *Micrococcus luteus*. These are strong agonists of the respective pathways but do not rapidly kill flies in which detection pathways are mutated, permitting us to assay gene expression at late times after infection. Fat body *Mef2* knockdown flies exhibited dramatically reduced induction of *AttA*, *CecA1*, *Def*, *Dpt*, *Dro*, *Drs*, *IM4*, and *Mtk* in response to this mixed infection (Figure 4, compare black bars to white bars). Again, this could be rescued by coexpression of wild-type *Mef2* (Figure 4, gray bars), and again, the few genes that were not completely rescued also exhibited reduced induction in flies carrying an inducible *Mef2-EnR* transgene (Figure S4A). This block was heterogeneous; at least one antimicrobial peptide, *Listericin*, was entirely unaffected (Figure 4). We observed similar results in animals that received single infections with either bacterium (Figure S4B). That both *Toll* and *imd* target genes were affected in response to an infection activating both pathways indicates that the role of MEF2 is not specific to either pathway.

MEF2 and TBP Physically Interact upon Infection to Bind AMP TATA Boxes

To clarify the requirement for *Mef2* in expression of its immune targets, we examined the requirement for individual MEF2 sites near antimicrobial peptides. We cloned putative regulatory regions (~1.5 kb 5′ to the ATG) from *Drosocin* and *Metchnikowin* upstream of enhanced GFP (eGFP). This region included NF-κB, DEAF-1, and GATA sites previously shown to regulate *Mtk* expression (Reed et al., 2008; Senger et al., 2004) as well as multiple putative MEF2 sites. In each case, a predicted MEF2 site overlapped the TATA box (*Dro* site 3 and *Mtk* site 2) (Figures 5A and S5). Individual putative MEF2 binding sites were mutated in these constructs, keeping the core TATA sequences intact; each *Mef2* mutant TATA sequence replicated a naturally occurring, functional TATA box not predicted to bind MEF2. We initially analyzed reporter activity of wild-type and mutant constructs in S2* cells treated with lipopolysaccharide (LPS) (commercial LPS contains sufficient peptidoglycan to potently activate the *imd* pathway [Kaneko et al., 2004]) (Figure S5). Both *Dro-GFP* and *Mtk-GFP* were strongly induced by LPS, as expected. Distal MEF2 sites were important for *Dro* reporter activity, but both *Mtk* and *Dro* reporters required the MEF2-TATA box for normal induction (Figure S5). To test in vivo responses to infection, we made transgenic flies carrying the same eight GFP reporters, each inserted into the AttP2 site. Uninfected, these animals exhibited weak or no GFP fluorescence, but *E. coli* injection induced strong fat body GFP activity in flies carrying the wild-type reporters

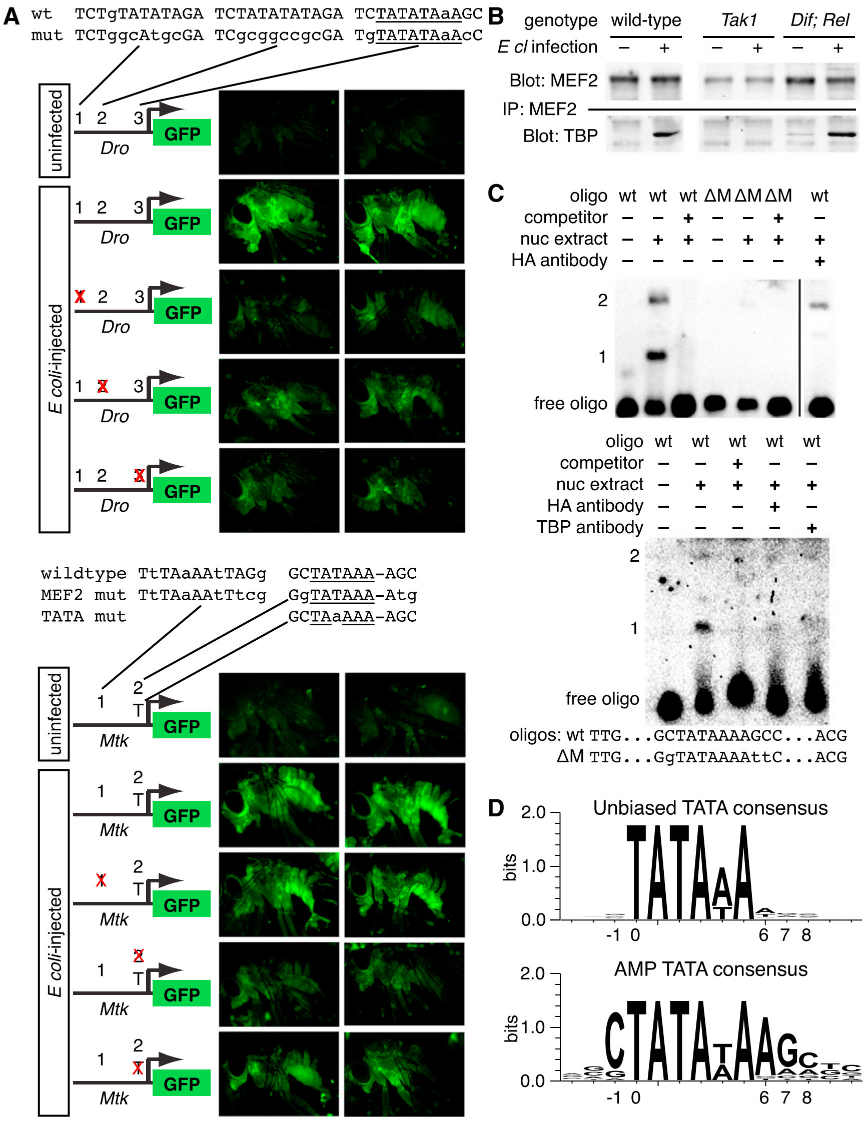

E

1st constraint (count) \ 2nd constraint	C -1	A +6	G +7	M +8	AG +67	AGM +678
	percentage of 1st-constraint TATA boxes also fitting 2nd constraint					
NTATAWANNN (890)	27.1	50.9	37.3	53.2	29.3	19.0
CTATAWANNN (241)	na	64.3**	46.1*	58.5	41.5**	29.1**
NTATAWAANN (453)	34.2**	na	57.6**	57.8	na	na
NTATAWANGN (332)	33.4*	78.6**	na	62.7**	na	na
NTATAWAAGN (261)	38.3**	na	na	64.8**	na	na
NTATAWAAGM (169)	41.4**	na	na	na	na	na

Figure 5. An Infection-Inducible MEF2-TBP Complex Binds AMP TATA Boxes

(A) Requirement for MEF2 and TBP sites in AMP regulation measured by eGFP fluorescence in transgenic flies carrying indicated reporters 8 hr after *E. coli* injection (general genotype: w^{1118}; ; AMP-*eGFP*[AttP2]/+). Two male flies are shown for each condition. In site maps, matches to the MEF2 consensus are capitalized, and matches to the TATA consensus are underlined.

(B) MEF2 and TBP coimmunoprecipitate from whole flies 3 hr after *E cloacae* infection; this association is eliminated in *Tak1* mutants, but not in *Dif; Rel* double mutants.

(C) MEF2 and TBP bind the *Mtk* TATA box. The top indicates EMSA with 40 bp oligonucleotides corresponding sequences surrounding the wild-type or non-MEF2-binding mutant (ΔM) TATA box. Nuclear extracts are all from cells expressing MEF2-HA. All lanes are from the same gel and used the same extracts. The bottom indicates a similar experiment, showing that anti-HA and anti-TBP antibodies can inhibit formation of the same complex.

(D) TATA box sequences from (top) unbiased 890-TATA sample; (bottom) 27 AMPs and AMP-like factors.

(E) Correlation of flanking residues in the 890-TATA sample. Asterisks indicate significant enrichment relative to unconstrained sample (*p < 0.01; **p < 0.001; assayed by binomial test).

See also Figure S5 and Tables S4 and S5.

(Figure 5A). The requirement for MEF2 sites in vivo was similar to that in vitro; in particular, the MEF2-TATA site was required in each case (Figure 5A).

We then tested whether MEF2 and TBP interacted directly by immunoprecipitating endogenous MEF2 from whole-fly lysates. In adult flies infected with the *imd* agonist *E. cloacae* or a mixture of *M. luteus* and *E. coli*, TBP coimmunoprecipitated with MEF2 (Figure 5B and data not shown). When stimulated by *E. cloacae*, this association required the *imd*-activated JNKKK *Tak1* but was unimpaired in *Dif; Rel* double mutants, suggesting a requirement for JNK signaling, but not NF-κB, in MEF2-TBP complex formation after Gram-negative infection (Figure 5B).

We next asked whether the MEF2-TBP complex could bind the putative MEF2-TATA site. The *Dro* MEF2-TATA site (TCTATATAAAGC) was a near-perfect match to the ideal MEF2A site as defined by SELEX (KCTAWWWWTAGM) (Pollock and Treisman, 1991), so we focused on the more divergent *Mtk* MEF2-TATA site (GCTATAAAAGC). This site eliminates one of the central A/T nucleotides in the MEF2 consensus, changing the spacing between the two putative half-sites. When assayed by EMSA, the *Mtk* core promoter was bound by nuclear extracts from S2R+ cells transfected with *Mef2*-HA, causing two distinct complexes to form: complex 1, at lower apparent molecular weight, was strong and consistently present, whereas the amount of complex 2 varied from experiment to experiment, though it was always less abundant than complex 1 (Figure 5C). Mutation of the putatively MEF2-binding C and G nucleotides flanking the TATA box eliminated both complexes. Complex 1 was also eliminated by preincubation of extracts with antibodies to either HA or TBP, indicating that this complex contained MEF2 and TBP together bound to the *Mtk* TATA sequence.

To test the generality of these observations, we examined the promoters of 30 antimicrobial peptides (AMPs) and other immune-inducible small peptides (Table S4). Of these 30 genes, 27 had a TATA box, defined as TATAWA with the initial T between −57 and −11 nucleotides relative to the annotated transcription start site (TSS). The consensus sequence defined by these 27 TATA boxes, CTATAWAAGM, is identical to the *Mtk* MEF2-TATA box (Figure 5D and Table S4). 17/27 AMP TATAs match this consensus; 3/27 instead match the *Dro* site, CTATA TAAAGC. The remaining seven TATA boxes include at least one nonconsensus nucleotide in the C...AGM flanks. The *Mtk* and *Dro*-like sites were found on peptides from many structural families, implying that this is not an ancestral sequence maintained by lack of counterselection. Conversely, the 13 metabolic genes shown in Figure 3A had a total of 27 TSSs; of these, 5 had TATA boxes, none of which matched the extended consensus associated with antimicrobial peptides.

We then examined roughly 15,000 unique promoters drawn from four databases of precisely mapped *Drosophila* TSSs (Hoskins et al., 2011; Ni et al., 2010; Rach et al., 2009; Schmid et al., 2006). This identified 890 TATA-containing promoters, with TATA boxes defined by the same criteria used for antimicrobial peptides (Table S5). The median TATA box position was 31 nucleotides upstream of the TSS (25–75 percentile range, 30–33 nucleotides) (Figure S5B). This set of TATA boxes contained 13 sequences that had been in our "training set" of AMPs and AMP-like peptides; these were not removed—and the other AMP TATAs were not added to this set—to avoid functionally biasing the represented genes. AMP promoters were more likely to contain a TATA box than those in the unbiased set (27/30 TATA-containing AMP promoters versus 890/15,000 TATA-containing promoters in the unbiased analysis). Unlike AMP TATA boxes, the 890 unbiased TATA boxes showed little nucleotide preference outside the core TATAWA, except a preference for A or T in the immediate 3′ position (Figure 5D).

If the nucleotides flanking the core TATAWA motif permit regulation of TATA usage via MEF2-TBP interaction, then the identities of these nucleotides should be correlated—the presence of a 5′ C should predict the presence of a 3′ AGM and vice versa. We examined the relationships among flanking nucleotide positions within the 890 unbiased TATA boxes. In each case, TATA boxes that fit the *Mtk*-like TATA consensus at one flanking nucleotide were more likely to match this consensus at other positions as well. For example, in the initial set, 27.1% of TATA boxes contained a C directly 5′ of the TATAWA core. Fixing the nucleotide 3′ of the TATAWA as A increased this representation to 34.2%, fixing the two 3′ nucleotides as AG gave a further increase to 38.3%, and fixing the three 3′ nucleotides as AGM increased the representation to 41.4% (Figure 5E).

We extracted from our set of TATA-containing promoters those matching the *Mtk*-like or *Dro*-like consensus. This gave 88 genes (Table S5). In addition to antimicrobial peptides, this list contained other infection-regulated genes and genes involved in responses to a variety of stresses. It also contained larval cuticular proteins, genes involved in sensory development or function, and 13 different uncharacterized small proteins with predicted secretory signals. The Gene Ontology terms "humoral immune response," "immune response," "defense response," and "antimicrobial humoral response" were overrepresented among genes with MEF2-TATA sequences as compared with the full set of 890 TATA-containing genes (Bonferroni-corrected $p < 0.01$).

These data together indicate that infection drives formation of a MEF2-TBP complex that can bind the TATA box of many antimicrobial peptides and is required for normal transcriptional induction of these genes. That the flanking nucleotide positions do not vary independently and the MEF2-TBP TATA box is overrepresented on immune genes both suggest that the MEF2-TBP TATA box represents a previously uncharacterized discrete gene regulatory element.

MEF2 Is Phosphorylated In Vivo at T20 to Regulate Association with TBP

The mechanisms by which the infection-inducible MEF2-TBP complex might be regulated and its potential function in MEF2 regulation of metabolic targets were unclear. We returned to our microarray data to directly compare expression of the metabolic and immune genes we had identified as *Mef2* regulated. After bacterial infection, these genes were strikingly counterregulated; when the antimicrobial genes were induced, expression of the metabolic genes was lost. This was especially pronounced 3 and 6 hr after septic injury and in late-stage mycobacterial infection (Figure 6A). This observation suggested that MEF2 modification might underlie a choice between immune and metabolic targets.

MEF2 activity can be regulated by several mechanisms, including phosphorylation by p38 MAP kinase, a calcineurin-regulated acetylation/sumoylation switch, and an indirect mechanism involving phosphorylation-regulated recruitment of class II histone deacetylases (Berdeaux et al., 2007; Han et al., 1997; Shalizi et al., 2006; Zhang et al., 2002). In agreement with previous work (Chen et al., 2010; Dijkers and O'Farrell, 2007), neither p38 nor calcineurin significantly altered immune competence in adult flies (data not shown).

MEF2 phosphorylation at T20, within the DNA-binding domain, can promote binding of MEF2 to a consensus MEF2-dimer binding sequence that does not contain a central TATAWA (Wang et al., 2005). The T20 region is conserved in *Drosophila*, *C. elegans*, and all mouse and human MEF2 homologs, and it matches a crude consensus site for AKT and p70 S6 kinase (Figure 6B). Using a phosphomotif antibody (Moritz et al., 2010), we found that MEF2 T20 phosphorylation was detectable in extracts from whole-adult *Drosophila* and was reduced in flies that had received weak or strong immune challenges (PBS injection or mixed infection with *M. luteus* and *E. coli*, respectively) (Figure 6C).

To test the relevance of this phosphorylation in transcriptional complex formation, we generated flies that inducibly expressed HA-tagged wild-type MEF2, nonphosphorylatable MEF2 (T20A), or phosphomimetic MEF2 (T20E) specifically in the fat body. One week after eclosion, these flies were shifted from 18° to 29° to drive *Mef2*.HA* expression. Because all transgenes were inserted into the same site, *Mef2** constructs were expressed at similar levels (Figure S6A).

We tested whether T20 directly regulated the association of MEF2 with TBP by immunoprecipitating fat-body-expressed

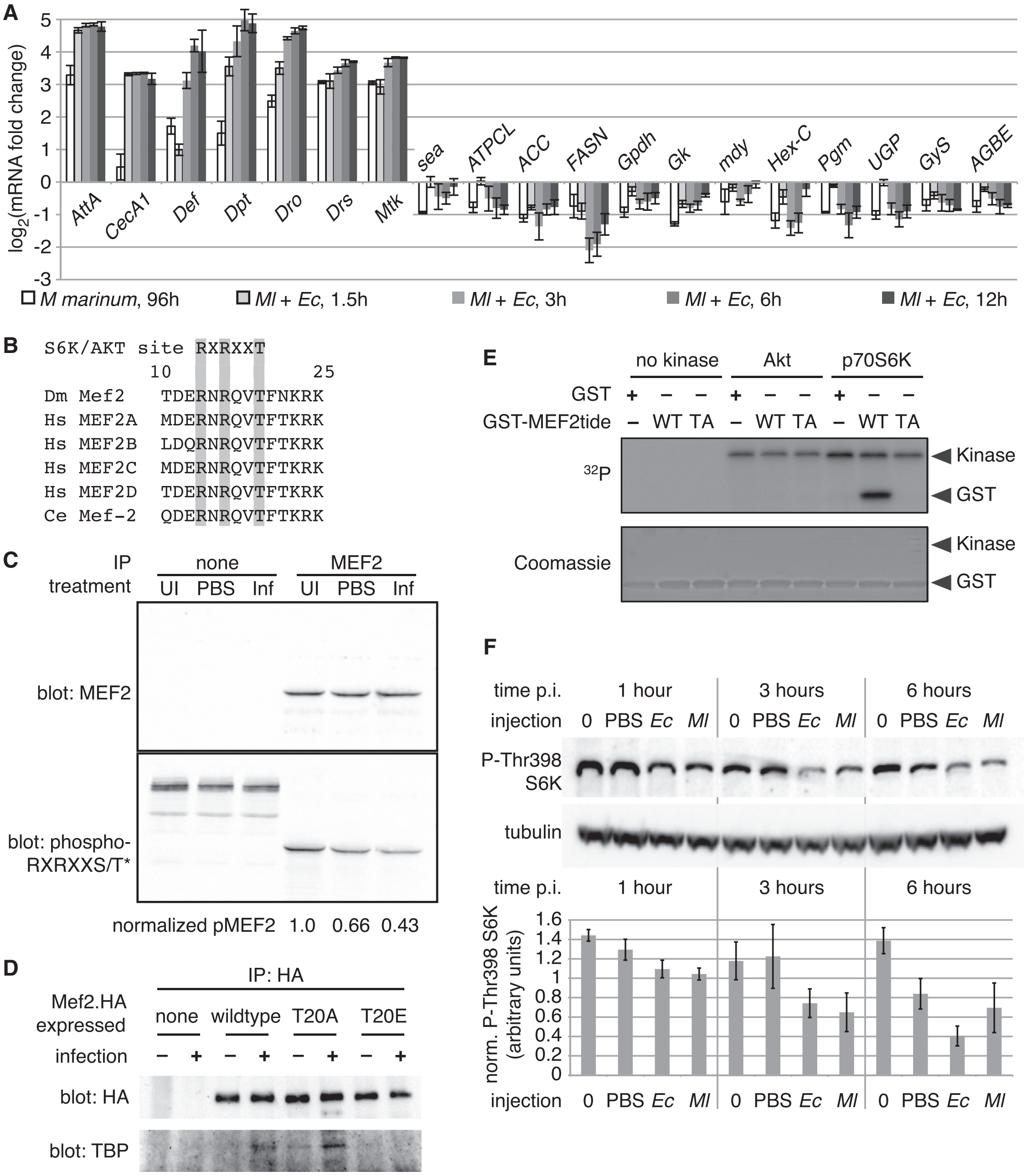

Figure 6. T20 Phosphorylation Regulates MEF2-TBP Association

(A) Counterregulation of *Mef2* targets after infection. Data are from microarrays discussed in text. Expression levels are normalized to uninfected controls. Values are mean ± SD.

(B) The *Mef2* T20 region is conserved in flies, humans, and *C. elegans* and matches an AKT/p70 S6 kinase consensus site.

(C) Endogenous MEF2 immunoprecipitated from whole flies is phosphorylated at T20; this phosphorylation is reduced by PBS injection or by infection with mixed *M. luteus* and *E. coli*. Top indicates immunoprecipitated MEF2 probed with anti-MEF2 antibody; below indicates the same blot probed with anti-phospho-RXRXXS/T* antibody; bottom indicates quantification of phospho-T20-MEF2 from the blot shown, normalized to total MEF2 and to the level in unmolested flies.

(D) Immunoprecipitation (IP) of inducible fat-body-expressed wild-type, T20A, or T20E MEF2.HA from flies either uninfected or infected 3 hr previously with mixed *E. coli* and *M. luteus*, probed with anti-HA (top) or for coimmunoprecipitated endogenous TBP (bottom). Genotype: w^{1118}/Y ; c564/+ ; UAS-*Mef2*.HA*/tub-*Gal80*^{ts}.

(E) T20 can be phosphorylated by p70 S6K in vitro. Purified GST or GST-MEF2tide fusions (wild-type or T20A) were incubated with ^{32}P-γ-ATP with or without recombinant kinases and resolved by SDS-PAGE. Top indicates ^{32}P autoradiogram; bottom indicates Coomassie blue stain.

(F) p70 S6K Thr398 phosphorylation is reduced by infection. Top indicates representative western on whole-fly lysates probed with the antibodies indicated; bottom indicates quantification of phospho-S6K normalized to tubulin from three independent experiments. Values are mean ± SD. Infections: *Ec* = *E. coli* (Gram-negative *imd* agonist); *Ml* = *M. luteus* (Gram-positive *Toll* agonist). Uninjected animals were anaesthetized alongside injected animals but were otherwise unmolested.

See also Figure S6.

MEF2*.HA either before or 3 hr after mixed infection. As was seen for the endogenous protein, wild-type MEF2.HA associated with endogenous TBP only after infection (Figure 6D). Nonphosphorylatable MEF2 associated with TBP even in the absence of infection; this association was strengthened by infection, suggesting that other MEF2 or TBP modifications also contribute to complex formation. In contrast, phosphomimetic MEF2 was not observed to associate with TBP either before or after infection.

We wished to identify a kinase or kinases responsible for MEF2 T20 phosphorylation in the *Drosophila* fat body. Others have observed MEF2C T20 phosphorylation by protein kinase A (PKA) and AKT (Wang et al., 2005). As PKA activation inhibits glycogenesis and promotes lipolysis and as AKT activity is reduced by infection in *Drosophila*, we initially focused on AKT as a likely T20 kinase. However, we were unable to observe phosphorylation of this site by AKT in vitro (Figure 6E). Like AKT, p70 S6 kinase (S6K) is broadly anabolic in its effects and is activated by nutrient signals. S6K shares with AKT its preference for substrates with arginine at −5 and −3 (Alessi et al., 1996). T20 was efficiently phosphorylated by recombinant S6K in vitro (Figure 6E), and systemic S6K activation (measured by phosphorylation at T398, corresponding to T389 in human S6K) was reduced in flies that had received infections with *Toll* or *imd* agonists (Figure 6F). A similar effect was seen during the last 3 days of life in flies infected with *M. marinum* (Figure S6B). S6K is thus a plausible in vivo MEF2 T20 kinase and may be responsible for promoting expression of the enzymes of lipogenesis and glycogenesis in healthy, well-fed animals.

T20 Regulates the Ability of MEF2 to Promote Distinct Target Genes and Physiologies

Together, these data suggested that loss of MEF2 T20 phosphorylation might cause the loss of anabolic transcripts seen after infection and that loss of T20 phosphorylation might be permissive for antimicrobial peptide expression. To test this model, we measured expression of immune and metabolic MEF2 targets in uninfected and infected animals inducibly expressing wild-type *Mef2*, *Mef2*^{T20E}, *Mef2*^{T20A}, or driver-only controls. In most cases, overexpression of wild-type MEF2 had small effects on either the loss of metabolic transcripts or the induction of antimicrobial peptides after infection when compared to the effects of infection in driver-only controls (Figures 7A and S7). Expression of nonphosphorylatable MEF2 enhanced expression of antimicrobial peptides (by as much as 6-fold, in the case of *Defensin*) after either *M. luteus* or *E. cloacae* infection but had little effect on most metabolic genes. Conversely, expression of phosphomimetic MEF2 had little general effect on antimicrobial peptides and did not counteract the loss of most anabolic transcripts after *M. luteus* infection but rescued levels of anabolic transcripts after *E. cloacae* infection.

Together, these data indicate that T20 phosphorylation can regulate the association of MEF2 with TBP, switching MEF2 between metabolic and immune functions. The loss of metabolic gene expression driven by *imd* pathway activation (typically caused by Gram-negative bacterial infection) stems, at least in part, from the loss of T20-phosphorylated MEF2.

DISCUSSION

Here, we identify *Mef2* as a factor critical for energy storage and the inducible immune response in the *Drosophila* fat body. Many infection-induced antimicrobial peptides depend on *Mef2* for normal expression. In consequence, flies lacking *Mef2* activity in the fat body are severely immunocompromised against a variety of infections. *Mef2* sites are also associated with genes encoding key enzymes of anabolism, and *Mef2* is required for normal expression of these genes; consequently, flies lacking *Mef2* function in the fat body exhibit striking reductions in the total levels of triglyceride and glycogen. These two groups of target genes are counterregulated during infection; the anabolic targets of *Mef2* are reduced in expression when antimicrobial peptides are induced. We show that fat body MEF2 can exist in two states with distinct physiological activities. In uninfected animals, MEF2 is phosphorylated at T20 and can promote the expression of its metabolic targets. In infected animals, T20 is dephosphorylated, and MEF2 associates with the TATA-binding protein to bind a compound MEF2-TATA sequence found in the core promoters of antimicrobial peptides. The loss of T20-phosphorylated MEF2 promotes the loss of anabolic transcripts in flies with Gram-negative bacterial infection. These data, taken together, suggest that the central role of MEF2 in promoting fat body anabolism and immune activity reflects a switch between distinct transcriptional states regulated, at least in part, by differential affinity for TBP determined by T20 phosphorylation (Figure 7B).

The signaling mechanisms regulating T20 phosphorylation and MEF2-TBP association are clearly of critical importance. The ability of p70 S6K to phosphorylate this residue is congruent with the ability of S6K to enhance anabolism and repress catabolism in response to nutrient signals (Laplante and Sabatini, 2012). However, others have shown T20 phosphorylation by PKA, suggesting that T20 phosphorylation may be regulated by more than one pathway in vivo (Wang et al., 2005). The role of TAK1 may be similarly complex. TAK1 is required for formation of the MEF2-TBP complex upon Gram-negative infection, but this effect may be indirect. For example, reduced S6K phosphorylation after infection may result from insulin resistance driven by TAK1 via JNK (Chen et al., 2002; Hirosumi et al., 2002). TAK1-dependent JNK activation is required for normal AMP induction in vivo (Delaney et al., 2006), but it remains possible that some novel pathway is the critical connection between TAK1 and MEF2-TBP complex formation.

In mammals, in addition to hematopoietic roles, *Mef2c* regulates B cell proliferation upon antigen stimulation (Khiem et al., 2008; Wilker et al., 2008), and *Mef2d* regulates IL2 and IL10 in T cells (Liopeta et al., 2009; Pan et al., 2004). The possibility that *Mef2* family proteins might be important direct activators of innate responses has not previously been examined. We show that *Mef2* is a core transcriptional component of the innate immune response of the adult fly. Equally, vertebrate *Mef2* family proteins are critical regulators of muscle metabolism, activated by physical activity to promote expression of PGC-1α and the glucose transporter *Glut4* (Handschin et al., 2003; Thai et al., 1998; Wu et al., 2001). *Glut4* regulation by MEF2 is known in adipose tissue as well as in muscle (Thai et al.,

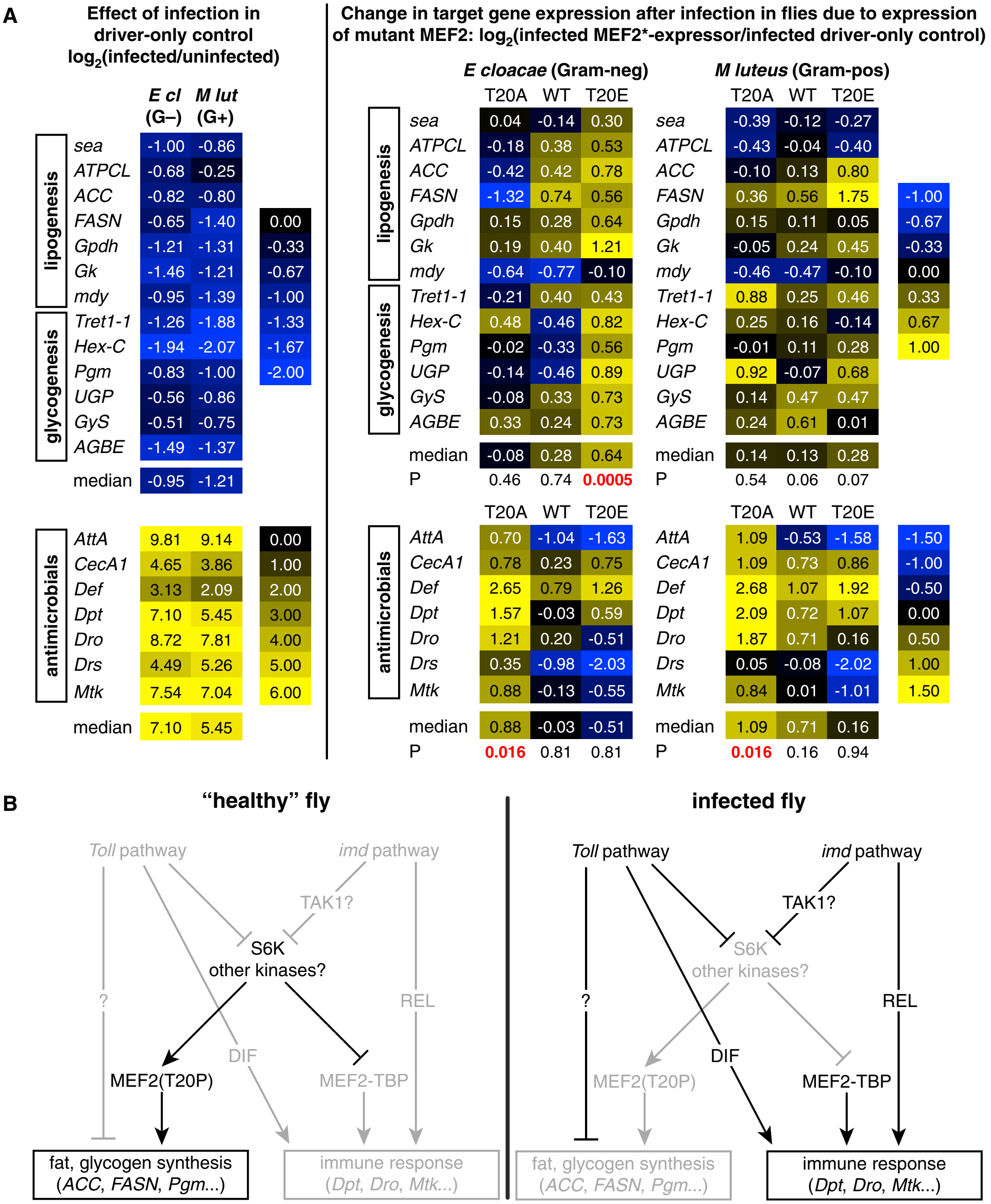

Figure 7. T20 Phosphorylation Regulates *Mef2* Target Choice in Infected Flies

(A) Effects of inducible fat body expression of wild-type, T20A, or T20E *Mef2* assayed by qRT-PCR. Genotypes are as in Figure 6D. *Mef2* expression was activated beginning 16 hr before infection. Left indicates the effect of infection in driver-only controls; right indicates changes due to misexpression of *Mef2* mutants. Overall significance of changes is assayed by Wilcoxon signed-rank test. All measurements are the mean of three biological replicates.

(B) Model. The position of *Tak1* is unresolved: though shown upstream of S6K inactivation, it could also promote MEF2-TBP interaction in parallel with S6K inactivation.

See also Figure S7.

1998); it is an intriguing possibility that MEF2 is as important a regulator of adipose metabolism in vertebrates as we show it to be in flies.

Infection-induced metabolic disruption leading to cachexia is present in vertebrates as well as in insects, most notoriously in Gram-negative sepsis and persistent bacterial infections such

as tuberculosis (Schwenk and Macallan, 2000; Tappy and Chioléro, 2007). Our data suggest that wasting seen after infection may be due, in part, to the requirement for MEF2 to serve different transcriptional functions in different conditions; the MEF2 immune-metabolic transcriptional switch may be a mechanistic constraint that forces the fly into metabolic pathophysiology in contexts of persistent immune activation. Alternatively, the loss of MEF2-driven anabolic transcripts due to infection may be productive, either by altering systemic energy usage or by increasing the production or release of one or more antimicrobial metabolites. Recent work has highlighted a distinction between "resistance" type immune mechanisms, in which the host attempts to eradicate an invading organism, and "tolerance" type mechanisms, in which the host response is oriented toward reducing the damage done by infection (Schneider and Ayres, 2008). The distinct metabolic and immune requirements for MEF2, combined with the obligation on the part of the host to raise some measure of resistance to systemic infection, may limit the achievable level of tolerance in persistent infections.

EXPERIMENTAL PROCEDURES

Fly Culture, Stocks, and Infections

Flies were maintained at 25°, 60% relative humidity, and on food containing 10% w/v Brewer's yeast, 8% fructose, 2% polenta, and 0.8% agar. Adult males were collected soon after eclosion and transferred to fresh food to age prior to treatment. All phenotypes shown were observed with VDRC *Mef2*-IR lines 15549 and 15550, which gave essentially identical results.

5- to 10-day-old adult male flies were injected with 50 nl of bacterial suspension or vehicle as described (Dionne et al., 2003). For survival, dead flies were counted at 24 hr intervals or more frequently. Survival analyses were repeated at least three times, always showing qualitatively similar results. Minimum cohort size was 20 flies.

Antimicrobial peptide reporter expression was assayed in live flies using a Leica M205 FA. Photographs were taken and processed using identical settings.

Microarrays

Experimental aspects of array analysis have been described (De Gregorio et al., 2001, 2002; Dionne et al., 2006). Data were reanalyzed using the BioConductor suite in R.

Clustering of Probes, Binding-Site Predictions, and Functional Predictions

We analyzed only probes significantly regulated (fold change > 1.5, $p < 0.01$) in end-stage *M. marinum* samples (Dionne et al., 2006). Probes were clustered by multiple rounds of fuzzy c-means clustering using MFuzz (Futschik and Carlisle, 2005).

For each cluster, extended gene regions (gene sequence including introns and UTRs, plus 2,000 bases flanking sequence) were analyzed with CLOVER (Frith et al., 2004), using *Drosophila* chr 3R as background sequence.

Binding sites were tested against 20 false clusters of genes (genes selected randomly from those represented on the Affymetrix *Drosophila* Genome 1 array and treated the same as true clusters). Sites predicted to regulate more than two false clusters were eliminated from consideration.

Functional predictions were from Generic GO Term Finder (http://go.princeton.edu/cgi-bin/GOTermFinder).

Biochemistry

EMSA, immunoprecipitation, kinase assays, and western blots were performed according to standard protocols as described in the Extended Experimental Procedures.

Quantitative Real-Time RT-PCR

Performed as previously described (Clark et al., 2011; Dionne et al., 2006). All data shown were the result of three to six biological replicates. Oligonucleotides are listed in Table S6.

ACCESSION NUMBERS

The ArrayExpress accession number for the *M. marinum* microarray data reported in this paper is E-MEXP-2510.

SUPPLEMENTAL INFORMATION

Supplemental Information includes Extended Experimental Procedures, seven figures, and six tables and can be found with this article online at http://dx.doi.org/10.1016/j.cell.2013.09.007.

ACKNOWLEDGMENTS

We thank the Vienna *Drosophila* RNAi Center, the Bloomington *Drosophila* Stock Center, Michael Taylor, Nazif Alic, Petros Ligoxygakis, Hanh Nguyen, and Richard Cripps for flies; Eileen Furlong and Bruce Paterson for antibodies; and Neal Silverman for peptidoglycan. Karen Liu, Vince Dionne, Brian Stramer, Thiago Carvalho, QueeLim Ch'ng, Joe Bateman, Rich Freiman, Mimi Shirasu-Hiza, David Schneider, and members of the Dionne, Stramer, and Geissmann laboratories provided essential discussion and comments. We would like to acknowledge funding from NIDDK (R01DK092590 to R.B.), NIAMS (R01AR059847 to R.B.), NIA (R01AG040288 and R01AG037514 to D.W.W.), and Ellison Medical Foundation (AG-NS-0470-08 to D.W.W.). Work in the Dionne lab was supported by grants from the BBSRC (BB/E02128X/1) and the Wellcome Trust (WT085119MA).

Received: August 24, 2012
Revised: July 8, 2013
Accepted: September 5, 2013
Published: September 26, 2013

REFERENCES

Alessi, D.R., Caudwell, F.B., Andjelkovic, M., Hemmings, B.A., and Cohen, P. (1996). Molecular basis for the substrate specificity of protein kinase B; comparison with MAPKAP kinase-1 and p70 S6 kinase. FEBS Lett. *399*, 333–338.

Becker, T., Loch, G., Beyer, M., Zinke, I., Aschenbrenner, A.C., Carrera, P., Inhester, T., Schultze, J.L., and Hoch, M. (2010). FOXO-dependent regulation of innate immune homeostasis. Nature *463*, 369–373.

Berdeaux, R., Goebel, N., Banaszynski, L., Takemori, H., Wandless, T., Shelton, G.D., and Montminy, M. (2007). SIK1 is a class II HDAC kinase that promotes survival of skeletal myocytes. Nat. Med. *13*, 597–603.

Blanchard, F.J., Collins, B., Cyran, S.A., Hancock, D.H., Taylor, M.V., and Blau, J. (2010). The transcription factor Mef2 is required for normal circadian behavior in Drosophila. J. Neurosci. *30*, 5855–5865.

Bour, B.A., O'Brien, M.A., Lockwood, W.L., Goldstein, E.S., Bodmer, R., Taghert, P.H., Abmayr, S.M., and Nguyen, H.T. (1995). Drosophila MEF2, a transcription factor that is essential for myogenesis. Genes Dev. *9*, 730–741.

Chambers, M.C., Song, K.H., and Schneider, D.S. (2012). Listeria monocytogenes infection causes metabolic shifts in Drosophila melanogaster. PLoS ONE *7*, e50679.

Chen, W., White, M.A., and Cobb, M.H. (2002). Stimulus-specific requirements for MAP3 kinases in activating the JNK pathway. J. Biol. Chem. *277*, 49105–49110.

Chen, J., Xie, C., Tian, L., Hong, L., Wu, X., and Han, J. (2010). Participation of the p38 pathway in Drosophila host defense against pathogenic bacteria and fungi. Proc. Natl. Acad. Sci. USA *107*, 20774–20779.

Chintapalli, V.R., Wang, J., and Dow, J.A. (2007). Using FlyAtlas to identify better Drosophila melanogaster models of human disease. Nat. Genet. *39*, 715–720.

Clark, R.I., Woodcock, K.J., Geissmann, F., Trouillet, C., and Dionne, M.S. (2011). Multiple TGF-β superfamily signals modulate the adult Drosophila immune response. Curr. Biol. *21*, 1672–1677.

De Gregorio, E., Spellman, P.T., Rubin, G.M., and Lemaitre, B. (2001). Genome-wide analysis of the Drosophila immune response by using oligonucleotide microarrays. Proc. Natl. Acad. Sci. USA *98*, 12590–12595.

De Gregorio, E., Spellman, P.T., Tzou, P., Rubin, G.M., and Lemaitre, B. (2002). The Toll and Imd pathways are the major regulators of the immune response in Drosophila. EMBO J. *21*, 2568–2579.

Delaney, J.R., Stöven, S., Uvell, H., Anderson, K.V., Engström, Y., and Mlodzik, M. (2006). Cooperative control of Drosophila immune responses by the JNK and NF-kappaB signaling pathways. EMBO J. *25*, 3068–3077.

DiAngelo, J.R., Bland, M.L., Bambina, S., Cherry, S., and Birnbaum, M.J. (2009). The immune response attenuates growth and nutrient storage in Drosophila by reducing insulin signaling. Proc. Natl. Acad. Sci. USA *106*, 20853–20858.

Dijkers, P.F., and O'Farrell, P.H. (2007). Drosophila calcineurin promotes induction of innate immune responses. Curr. Biol. *17*, 2087–2093.

Dionne, M.S., Ghori, N., and Schneider, D.S. (2003). Drosophila melanogaster is a genetically tractable model host for Mycobacterium marinum. Infect. Immun. *71*, 3540–3550.

Dionne, M.S., Pham, L.N., Shirasu-Hiza, M., and Schneider, D.S. (2006). Akt and FOXO dysregulation contribute to infection-induced wasting in Drosophila. Curr. Biol. *16*, 1977–1985.

Frith, M.C., Fu, Y., Yu, L., Chen, J.F., Hansen, U., and Weng, Z. (2004). Detection of functional DNA motifs via statistical over-representation. Nucleic Acids Res. *32*, 1372–1381.

Futschik, M.E., and Carlisle, B. (2005). Noise-robust soft clustering of gene expression time-course data. J. Bioinform. Comput. Biol. *3*, 965–988.

Gottar, M., Gobert, V., Matskevich, A.A., Reichhart, J.M., Wang, C., Butt, T.M., Belvin, M., Hoffmann, J.A., and Ferrandon, D. (2006). Dual detection of fungal infections in Drosophila via recognition of glucans and sensing of virulence factors. Cell *127*, 1425–1437.

Han, J., Jiang, Y., Li, Z., Kravchenko, V.V., and Ulevitch, R.J. (1997). Activation of the transcription factor MEF2C by the MAP kinase p38 in inflammation. Nature *386*, 296–299.

Handschin, C., Rhee, J., Lin, J., Tarr, P.T., and Spiegelman, B.M. (2003). An autoregulatory loop controls peroxisome proliferator-activated receptor gamma coactivator 1alpha expression in muscle. Proc. Natl. Acad. Sci. USA *100*, 7111–7116.

Hedengren, M., Asling, B., Dushay, M.S., Ando, I., Ekengren, S., Wihlborg, M., and Hultmark, D. (1999). Relish, a central factor in the control of humoral but not cellular immunity in Drosophila. Mol. Cell *4*, 827–837.

Hirosumi, J., Tuncman, G., Chang, L., Görgün, C.Z., Uysal, K.T., Maeda, K., Karin, M., and Hotamisligil, G.S. (2002). A central role for JNK in obesity and insulin resistance. Nature *420*, 333–336.

Hoskins, R.A., Landolin, J.M., Brown, J.B., Sandler, J.E., Takahashi, H., Lassmann, T., Yu, C., Booth, B.W., Zhang, D., Wan, K.H., et al. (2011). Genome-wide analysis of promoter architecture in Drosophila melanogaster. Genome Res. *21*, 182–192.

Hotamisligil, G.S. (2006). Inflammation and metabolic disorders. Nature *444*, 860–867.

Hrdlicka, L., Gibson, M., Kiger, A., Micchelli, C., Schober, M., Schöck, F., and Perrimon, N. (2002). Analysis of twenty-four Gal4 lines in Drosophila melanogaster. Genesis *34*, 51–57.

Kaneko, T., Goldman, W.E., Mellroth, P., Steiner, H., Fukase, K., Kusumoto, S., Harley, W., Fox, A., Golenbock, D., and Silverman, N. (2004). Monomeric and polymeric gram-negative peptidoglycan but not purified LPS stimulate the Drosophila IMD pathway. Immunity *20*, 637–649.

Khiem, D., Cyster, J.G., Schwarz, J.J., and Black, B.L. (2008). A p38 MAPK-MEF2C pathway regulates B-cell proliferation. Proc. Natl. Acad. Sci. USA *105*, 17067–17072.

Laplante, M., and Sabatini, D.M. (2012). mTOR signaling in growth control and disease. Cell *149*, 274–293.

Lemaitre, B., and Hoffmann, J. (2007). The host defense of Drosophila melanogaster. Annu. Rev. Immunol. *25*, 697–743.

Lilly, B., Zhao, B., Ranganayakulu, G., Paterson, B.M., Schulz, R.A., and Olson, E.N. (1995). Requirement of MADS domain transcription factor D-MEF2 for muscle formation in Drosophila. Science *267*, 688–693.

Liopeta, K., Boubali, S., Virgilio, L., Thyphronitis, G., Mavrothalassitis, G., Dimitracopoulos, G., and Paliogianni, F. (2009). cAMP regulates IL-10 production by normal human T lymphocytes at multiple levels: a potential role for MEF2. Mol. Immunol. *46*, 345–354.

Molkentin, J.D., Black, B.L., Martin, J.F., and Olson, E.N. (1995). Cooperative activation of muscle gene expression by MEF2 and myogenic bHLH proteins. Cell *83*, 1125–1136.

Moritz, A., Li, Y., Guo, A., Villén, J., Wang, Y., MacNeill, J., Kornhauser, J., Sprott, K., Zhou, J., Possemato, A., et al. (2010). Akt-RSK-S6 kinase signaling networks activated by oncogenic receptor tyrosine kinases. Sci. Signal. *3*, ra64.

Ni, T., Corcoran, D.L., Rach, E.A., Song, S., Spana, E.P., Gao, Y., Ohler, U., and Zhu, J. (2010). A paired-end sequencing strategy to map the complex landscape of transcription initiation. Nat. Methods *7*, 521–527.

Pan, F., Ye, Z., Cheng, L., and Liu, J.O. (2004). Myocyte enhancer factor 2 mediates calcium-dependent transcription of the interleukin-2 gene in T lymphocytes: a calcium signaling module that is distinct from but collaborates with the nuclear factor of activated T cells (NFAT). J. Biol. Chem. *279*, 14477–14480.

Pollock, R., and Treisman, R. (1991). Human SRF-related proteins: DNA-binding properties and potential regulatory targets. Genes Dev. *5*(12A), 2327–2341.

Rach, E.A., Yuan, H.Y., Majoros, W.H., Tomancak, P., and Ohler, U. (2009). Motif composition, conservation and condition-specificity of single and alternative transcription start sites in the Drosophila genome. Genome Biol. *10*, R73.

Reed, D.E., Huang, X.M., Wohlschlegel, J.A., Levine, M.S., and Senger, K. (2008). DEAF-1 regulates immunity gene expression in Drosophila. Proc. Natl. Acad. Sci. USA *105*, 8351–8356.

Rynes, J., Donohoe, C.D., Frommolt, P., Brodesser, S., Jindra, M., and Uhlirova, M. (2012). Activating transcription factor 3 regulates immune and metabolic homeostasis. Mol. Cell. Biol. *32*, 3949–3962.

Schmid, C.D., Perier, R., Praz, V., and Bucher, P. (2006). EPD in its twentieth year: towards complete promoter coverage of selected model organisms. Nucleic Acids Res. *34*(Database issue), D82–D85.

Schneider, D.S., and Ayres, J.S. (2008). Two ways to survive infection: what resistance and tolerance can teach us about treating infectious diseases. Nat. Rev. Immunol. *8*, 889–895.

Schwenk, A., and Macallan, D.C. (2000). Tuberculosis, malnutrition and wasting. Curr. Opin. Clin. Nutr. Metab. Care *3*, 285–291.

Senger, K., Armstrong, G.W., Rowell, W.J., Kwan, J.M., Markstein, M., and Levine, M. (2004). Immunity regulatory DNAs share common organizational features in Drosophila. Mol. Cell *13*, 19–32.

Shalizi, A., Gaudillière, B., Yuan, Z., Stegmüller, J., Shirogane, T., Ge, Q., Tan, Y., Schulman, B., Harper, J.W., and Bonni, A. (2006). A calcium-regulated MEF2 sumoylation switch controls postsynaptic differentiation. Science *311*, 1012–1017.

Tappy, L., and Chioléro, R. (2007). Substrate utilization in sepsis and multiple organ failure. Crit. Care Med. *35*(Suppl), S531–S534.

Thai, M.V., Guruswamy, S., Cao, K.T., Pessin, J.E., and Olson, A.L. (1998). Myocyte enhancer factor 2 (MEF2)-binding site is required for GLUT4 gene expression in transgenic mice. Regulation of MEF2 DNA binding activity in insulin-deficient diabetes. J. Biol. Chem. *273*, 14285–14292.

Tracey, K.J., and Cerami, A. (1994). Tumor necrosis factor: a pleiotropic cytokine and therapeutic target. Annu. Rev. Med. *45*, 491–503.

Vallerie, S.N., and Hotamisligil, G.S. (2010). The role of JNK proteins in metabolism. Sci. Transl. Med. *2*, rv5.

Van den Berghe, G. (2002). Neuroendocrine pathobiology of chronic critical illness. Crit. Care Clin. *18*, 509–528.

Wang, X., Tang, X., Li, M., Marshall, J., and Mao, Z. (2005). Regulation of neuroprotective activity of myocyte-enhancer factor 2 by cAMP-protein kinase A signaling pathway in neuronal survival. J. Biol. Chem. *280*, 16705–16713.

Wilker, P.R., Kohyama, M., Sandau, M.M., Albring, J.C., Nakagawa, O., Schwarz, J.J., and Murphy, K.M. (2008). Transcription factor Mef2c is required for B cell proliferation and survival after antigen receptor stimulation. Nat. Immunol. *9*, 603–612.

Wu, H., Rothermel, B., Kanatous, S., Rosenberg, P., Naya, F.J., Shelton, J.M., Hutcheson, K.A., DiMaio, J.M., Olson, E.N., Bassel-Duby, R., and Williams, R.S. (2001). Activation of MEF2 by muscle activity is mediated through a calcineurin-dependent pathway. EMBO J. *20*, 6414–6423.

Zhang, C.L., McKinsey, T.A., Chang, S., Antos, C.L., Hill, J.A., and Olson, E.N. (2002). Class II histone deacetylases act as signal-responsive repressors of cardiac hypertrophy. Cell *110*, 479–488.

EXTENDED EXPERIMENTAL PROCEDURES

Fly Infections, Metabolic Assays, and Stress Assays

5-10 day old adult male flies were injected with 50nl of bacterial suspension, or vehicle, as described (Dionne et al., 2003). For survival, dead flies were counted at 24 hr intervals or more frequently. Survival analyses were repeated at least three times, always showing qualitatively similar results. Minimum cohort size was 20 flies.

Enzymatic metabolic assays, thin-layer chromatography, and Oil Red O staining were performed as described (Al-Anzi and Zinn, 2010; Dionne et al., 2006; Gutierrez et al., 2007). For enzymatic assays, three male flies were homogenized in 75 μl TE with 0.1% Triton X-100. 10 μl of this lysate was used for glycogen, glucose and fat assays. Total glucose and trehalose was measured using liquid glucose oxidase reagent (Pointe Laboratories); holding the fly lysates at room-temperature for 5 min permits endogenous trehalase to cleave trehalose into two glucose equivalents, so that total circulating sugar is measured as glucose equivalents. To measure glycogen, this reagent was supplemented with 1 U/ml amyloglucosidase. This reaction was blanked against the glucose reaction. Triglyceride was measured by the method of McGowan et al. (1983). This reaction was blanked against a reaction without lipase.

For survival under hyperoxia, one-week-old male flies were placed in an 80% oxygen atmosphere, at room temperature. Normoxia controls were seven day old males removed to room temperature. Flies were flipped to fresh vials every 2-3 days throughout these experiments, and deaths were recorded every 12 hr. No deaths were seen in the normoxia control vials throughout the course of these experiments. Each experiment contained a minimum of 160 flies of each genotype in each condition.

For heat stress assays, male flies were aged as described above. One-week-old males were placed at 37°C and deaths were recorded every 1.5 hr. Each experiment contained a minimum of 160 flies of each genotype.

Clustering of Probes and Functional Predictions

We analyzed only probes significantly regulated ($|\log_2(\text{fold-change})| > 0.58$, $p < 0.01$) in end-stage *M marinum* samples (Dionne et al., 2006). This data set contains expression data for 823 probes on 79 arrays reflecting 29 biological conditions. These were subjected to three independent rounds of fuzzy c-means clustering using MFuzz with m = 1.05 and n = 36 (Futschik and Carlisle, 2005). Individual clustering runs were then analyzed to identify groups of probes that consistently clustered together: a cluster was defined as a group of probes that clustered together on two of three Mfuzz runs, with the further requirement that the probe have a membership value of at least 0.94 on both selecting runs. This gave 29 clusters (A-Z, AA, AB and AC). Cluster D was subdivided into D1 and D2 by hierarchical clustering of the individual genes using regulatory scores from the first round of CLOVER predictions. Functional predictions were from Generic GO Term Finder, < http://go.princeton.edu/cgi-bin/GOTermFinder >.

Binding Site Predictions

For each cluster, extended gene regions (gene sequence including introns and UTRs, plus 2000 bases of flanking sequence) were compiled into a single textfile. Where adjacent genes appeared in a single cluster, no sequences were duplicated. This was analyzed using CLOVER (Frith et al., 2004) with a pseudocount of 0.375, using *Drosophila* chromosome 3R (release 4) as background sequence. We excluded sites with p > 0.02.

Each transcription-factor binding site matrix was analyzed using the same parameters against 20 false clusters of genes (genes selected randomly from those represented on the Affymetrix *Drosophila* Genome 1 array and treated the same as the clusters above). Matrices that appeared frequently as regulators of these false clusters were eliminated from consideration.

Binding-site matrices were from four sources: Jaspar (Bryne et al., 2008), Transfac Public (Matys et al., 2003), Daniel Pollard's compilation of the DNase-fingerprinted sites in RedFly (Halfon et al., 2008), and directly from the primary literature (only these are listed in Table S2). Matrices corresponding to fewer than 15 identified sequences were expressed as percentage counts to eliminate noise due to the pseudocount. In some cases, we have used two different versions of the same matrix, one with low-information flanking nucleotides removed; the short version is referred to as "crop" or "trim" and an "a" is appended to the identifier. These are the only cases in which a given experimentally-derived matrix is represented more than once.

Sequence logos were created using WebLogo 3 with the background composition set to *Drosophila melanogaster* (Crooks et al., 2004).

Microbial Methods

Mycobacterium marinum was cultured as described (Dionne et al., 2006). *E. coli* (DH5α), *E cloacae* (NCTC 10005), and *M luteus* (clinical isolate, gift of W. Wade) were cultured overnight shaking at 37° in LB without antibiotics. *L monocytogenes* (NCTC 7973) was cultured overnight standing at 37° in BHI without antibiotics. *C albicans* was cultured overnight shaking at 30° in YT without antibiotics. Microbes were resuspended in sterile PBS for injection.

Immunoprecipitation and Western Blots

Western blots on whole-fly lysates were performed by smashing 3 adult male flies in 75 μl 2x Laemmli SDS-PAGE buffer with DTT; 5 μl of this lysate were used per gel lane.

For immunoprecipitation, whole-fly lysates were prepared by smashing 20-50 adult male *Drosophila* in ice-cold 25 mM HEPES pH 7.6, 100 mM KCl, 1% Nonidet P-40, 0.1 mM EDTA, 12.5 mM $MgCl_2$, 10% glycerol, 0.1 mM DTT, supplemented with phosphatase and

protease inhibitors (Roche). These extracts were then incubated overnight at 4° with protein-A or G sepharose or dynabeads. Anti-MEF2 (rabbit, from E Furlong) or anti-HA (mouse, Covance 16B12) were added in the morning and permitted to bind for at least one hour at 4°. Beads were then washed extensively in IP lysis buffer and finally resuspended in 2x Laemmli SDS-PAGE buffer with DTT for detection by Western blot.

Antibodies were from Cell Signaling (phospho-*Drosophila* S6 Kinase 9209, phospho-AKT substrate 10001 (Moritz et al., 2010), secondary antibodies 7076, 7074 and 3678), Developmental Studies Hybridoma Bank (α-tubulin 12G10), Sigma (TBP T1827), Covance (HA 16B12), Bethyl (HRP-anti-mouse λ A90-121P) and E. Furlong and B. Paterson (*Drosophila* MEF2) (Lilly et al., 1995; Sandmann et al., 2006).

Antimicrobial Peptide Reporters and UAS-*Mef2** Constructs

Mtk and *Dro* enhancer/promoters were amplified from *Drosophila* genomic DNA and cloned upstream of eGFP in the vector pCS2P-eGFP-X/P, simultaneously eliminating the sCMV promoter. Mutants were generated using DpnI mutagenesis, sequenced, and a fragment containing the mutation was cloned into the parental plasmid. Site locations, relative to initiator ATG: *Dro* 1, −1240 to −1229; *Dro* 2, −947 to −936; *Dro* 3, −66 to −54; *Mtk* 1, −804 to −793; *Mtk* 2 and T, −62 to −51. Primers are in Table S4. S2* cells (Silverman et al., 2000) were transfected using Effectene (QIAGEN). 24 hr later, they were treated with 1 μm 20-hydroxyecdysone (Sigma); 24 hr after this, medium was supplemented with 10 μg/ml LPS (Sigma, O55:B5) or vehicle. GFP was assayed 6 (vehicle, LPS) and 28 (LPS) hours later.

For UAS-*Mef2** constructs, *Mef2*-RB was PCR cloned from adult fly cDNA. Mutants were generated using DpnI mutagenesis, sequenced, and cloned into pUAST-AttB (Bischof et al., 2007). These were inserted into the AttP2 and AttP40 landing sites (Groth et al., 2004).

For reporter flies, AMP-eGFP constructs were cloned into pUAST-AttB, simultaneously eliminating the UAS, and inserted into the AttP2 landing site.

EMSA

The LightShift Chemiluminescent EMSA Kit (Thermo Scientific) was used according to the manufacturer's instructions, with the following modifications. 2 femtomoles of double stranded oligo were mixed with 1-2 μg of nuclear extract and incubated 10 min at room temperature in 20 μl of 10 mM Tris pH 7.6, 75 mM KCl, 1 mM DTT, 2.5 mM $MgCl_2$, 5% glycerol, 1 μg poly(dI-dC) acid sodium salt (Sigma). 5 pm unlabeled double-stranded oligo was used as a competitor. For supershift, nuclear extracts were preincubated with antibodies in binding buffer for 1 hr at 4° before the addition of biotinylated oligos; incubation with a control anti-GFP antibody did not disrupt observed binding (data not shown). EMSA reactions were run on 5% native acrylamide gel, containing 1% glycerol.

Kinase Assays

GST-MEF2tide-WT and GST-MEF2tide-T20A fusion protein constructs encoding GST, a glycine-rich linker and amino acids 14-24 of MEF2 were constructed using the Gibson Assembly Kit (NEB) in pGEX-KG and confirmed by sequencing. GST (from pGEX4T1) and GST-MEF2tide were purified from *E. coli* on Glutathione Sepharose-4B (GE Healthcare) and washed 3 times in kinase assay buffer (AKT: 50 mM Tris-HCl pH 7.5, 10 mM $MgCl_2$, 1 mM DTT; P70S6K: 50 mM Tris-HCl pH7.5, 10 mM MgOAc, 0.1 mM EGTA, 0.15% β-mercaptoethanol). 2 μg GST fusion protein was incubated with 0.1U recombinant AKT (Millipore, 14-276) or 0.02U recombinant activated p70S6 Kinase (T412E) (Millipore, 14-486) and 2 μCi γ-^{32}P-ATP in 1X buffer for 30 min at 30°C. Boiled reactions were resolved on 12.5% SDS-PAGE gels and stained with Coomassie Blue. Dried gels were exposed to autoradiography film.

SUPPLEMENTAL REFERENCES

Al-Anzi, B., and Zinn, K. (2010). Colorimetric measurement of triglycerides cannot provide an accurate measure of stored fat content in Drosophila. PLoS ONE *5*, e12353.

Bischof, J., Maeda, R.K., Hediger, M., Karch, F., and Basler, K. (2007). An optimized transgenesis system for Drosophila using germ-line-specific phiC31 integrases. Proc. Natl. Acad. Sci. USA *104*, 3312–3317.

Bryne, J.C., Valen, E., Tang, M.H., Marstrand, T., Winther, O., da Piedade, I., Krogh, A., Lenhard, B., and Sandelin, A. (2008). JASPAR, the open access database of transcription factor-binding profiles: new content and tools in the 2008 update. Nucleic Acids Res. *36*(Database issue), D102–D106.

Crooks, G.E., Hon, G., Chandonia, J.M., and Brenner, S.E. (2004). WebLogo: a sequence logo generator. Genome Res. *14*, 1188–1190.

Georgel, P., Naitza, S., Kappler, C., Ferrandon, D., Zachary, D., Swimmer, C., Kopczynski, C., Duyk, G., Reichhart, J.M., and Hoffmann, J.A. (2001). Drosophila immune deficiency (IMD) is a death domain protein that activates antibacterial defense and can promote apoptosis. Dev. Cell *1*, 503–514.

Groth, A.C., Fish, M., Nusse, R., and Calos, M.P. (2004). Construction of transgenic Drosophila by using the site-specific integrase from phage phiC31. Genetics *166*, 1775–1782.

Gutierrez, E., Wiggins, D., Fielding, B., and Gould, A.P. (2007). Specialized hepatocyte-like cells regulate Drosophila lipid metabolism. Nature *445*, 275–280.

Haenlin, M., Cubadda, Y., Blondeau, F., Heitzler, P., Lutz, Y., Simpson, P., and Ramain, P. (1997). Transcriptional activity of pannier is regulated negatively by heterodimerization of the GATA DNA-binding domain with a cofactor encoded by the u-shaped gene of Drosophila. Genes Dev. *11*, 3096–3108.

Halfon, M.S., Gallo, S.M., and Bergman, C.M. (2008). REDfly 2.0: an integrated database of cis-regulatory modules and transcription factor binding sites in Drosophila. Nucleic Acids Res. *36*(Database issue), D594–D598.

Hollenhorst, P.C., Shah, A.A., Hopkins, C., and Graves, B.J. (2007). Genome-wide analyses reveal properties of redundant and specific promoter occupancy within the ETS gene family. Genes Dev. *21*, 1882–1894.

Jaźwińska, A., Kirov, N., Wieschaus, E., Roth, S., and Rushlow, C. (1999). The Drosophila gene brinker reveals a novel mechanism of Dpp target gene regulation. Cell *96*, 563–573.

Johnson, K., Kirkpatrick, H., Comer, A., Hoffmann, F.M., and Laughon, A. (1999). Interaction of Smad complexes with tripartite DNA-binding sites. J. Biol. Chem. *274*, 20709–20716.

Jun, S., Wallen, R.V., Goriely, A., Kalionis, B., and Desplan, C. (1998). Lune/eye gone, a Pax-like protein, uses a partial paired domain and a homeodomain for DNA recognition. Proc. Natl. Acad. Sci. USA *95*, 13720–13725.

Kim, J., Johnson, K., Chen, H.J., Carroll, S., and Laughon, A. (1997). Drosophila Mad binds to DNA and directly mediates activation of vestigial by Decapentaplegic. Nature *388*, 304–308.

Kinzler, K.W., and Vogelstein, B. (1990). The GLI gene encodes a nuclear protein which binds specific sequences in the human genome. Mol. Cell. Biol. *10*, 634–642.

Knirr, S., and Frasch, M. (2001). Molecular integration of inductive and mesoderm-intrinsic inputs governs even-skipped enhancer activity in a subset of pericardial and dorsal muscle progenitors. Dev. Biol. *238*, 13–26.

Lee, H.H., and Frasch, M. (2000). Wingless effects mesoderm patterning and ectoderm segmentation events via induction of its downstream target sloppy paired. Development *127*, 5497–5508.

Ma, L., Robinson, L.N., and Towle, H.C. (2006). ChREBP*Mlx is the principal mediator of glucose-induced gene expression in the liver. J. Biol. Chem. *281*, 28721–28730.

Matys, V., Fricke, E., Geffers, R., Gössling, E., Haubrock, M., Hehl, R., Hornischer, K., Karas, D., Kel, A.E., Kel-Margoulis, O.V., et al. (2003). TRANSFAC: transcriptional regulation, from patterns to profiles. Nucleic Acids Res. *31*, 374–378.

McGowan, M.W., Artiss, J.D., Strandbergh, D.R., and Zak, B. (1983). A peroxidase-coupled method for the colorimetric determination of serum triglycerides. Clin. Chem. *29*, 538–542.

Nye, J.A., Petersen, J.M., Gunther, C.V., Jonsen, M.D., and Graves, B.J. (1992). Interaction of murine ets-1 with GGA-binding sites establishes the ETS domain as a new DNA-binding motif. Genes Dev. *6*, 975–990.

Pyrowolakis, G., Hartmann, B., Müller, B., Basler, K., and Affolter, M. (2004). A simple molecular complex mediates widespread BMP-induced repression during Drosophila development. Dev. Cell *7*, 229–240.

Sandmann, T., Jensen, L.J., Jakobsen, J.S., Karzynski, M.M., Eichenlaub, M.P., Bork, P., and Furlong, E.E. (2006). A temporal map of transcription factor activity: mef2 directly regulates target genes at all stages of muscle development. Dev. Cell *10*, 797–807.

Silverman, N., Zhou, R., Stöven, S., Pandey, N., Hultmark, D., and Maniatis, T. (2000). A Drosophila IkappaB kinase complex required for Relish cleavage and antibacterial immunity. Genes Dev. *14*, 2461–2471.

Urness, L.D., and Thummel, C.S. (1990). Molecular interactions within the ecdysone regulatory hierarchy: DNA binding properties of the Drosophila ecdysone-inducible E74A protein. Cell *63*, 47–61.

Vokes, S.A., Ji, H., McCuine, S., Tenzen, T., Giles, S., Zhong, S., Longabaugh, W.J., Davidson, E.H., Wong, W.H., and McMahon, A.P. (2007). Genomic characterization of Gli-activator targets in sonic hedgehog-mediated neural patterning. Development *134*, 1977–1989.

Xu, X., Yin, Z., Hudson, J.B., Ferguson, E.L., and Frasch, M. (1998). Smad proteins act in combination with synergistic and antagonistic regulators to target Dpp responses to the Drosophila mesoderm. Genes Dev. *12*, 2354–2370.

Yan, S.J., Gu, Y., Li, W.X., and Fleming, R.J. (2004). Multiple signaling pathways and a selector protein sequentially regulate Drosophila wing development. Development *131*, 285–298.

Yao, L.C., Phin, S., Cho, J., Rushlow, C., Arora, K., and Warrior, R. (2008). Multiple modular promoter elements drive graded brinker expression in response to the Dpp morphogen gradient. Development *135*, 2183–2192.

Yoshida, H., Haze, K., Yanagi, H., Yura, T., and Mori, K. (1998). Identification of the cis-acting endoplasmic reticulum stress response element responsible for transcriptional induction of mammalian glucose-regulated proteins. Involvement of basic leucine zipper transcription factors. J. Biol. Chem. *273*, 33741–33749.

Zhang, H., Levine, M., and Ashe, H.L. (2001). Brinker is a sequence-specific transcriptional repressor in the Drosophila embryo. Genes Dev. *15*, 261–266.

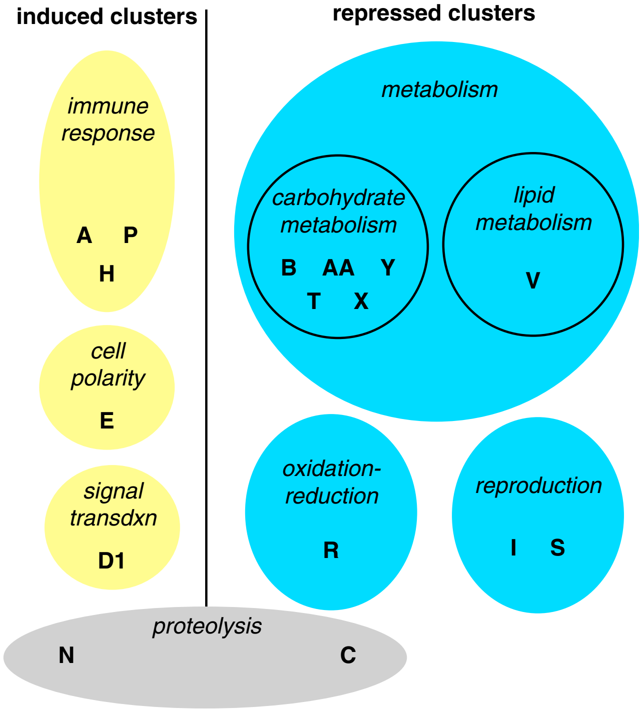

Figure S1. Cluster GO Term Enrichment, Related to Figure 1
Gene Ontology Process terms significantly (Bonferroni-corrected $p < 0.02$) enriched in each cluster. Clusters with no enriched terms are not shown.

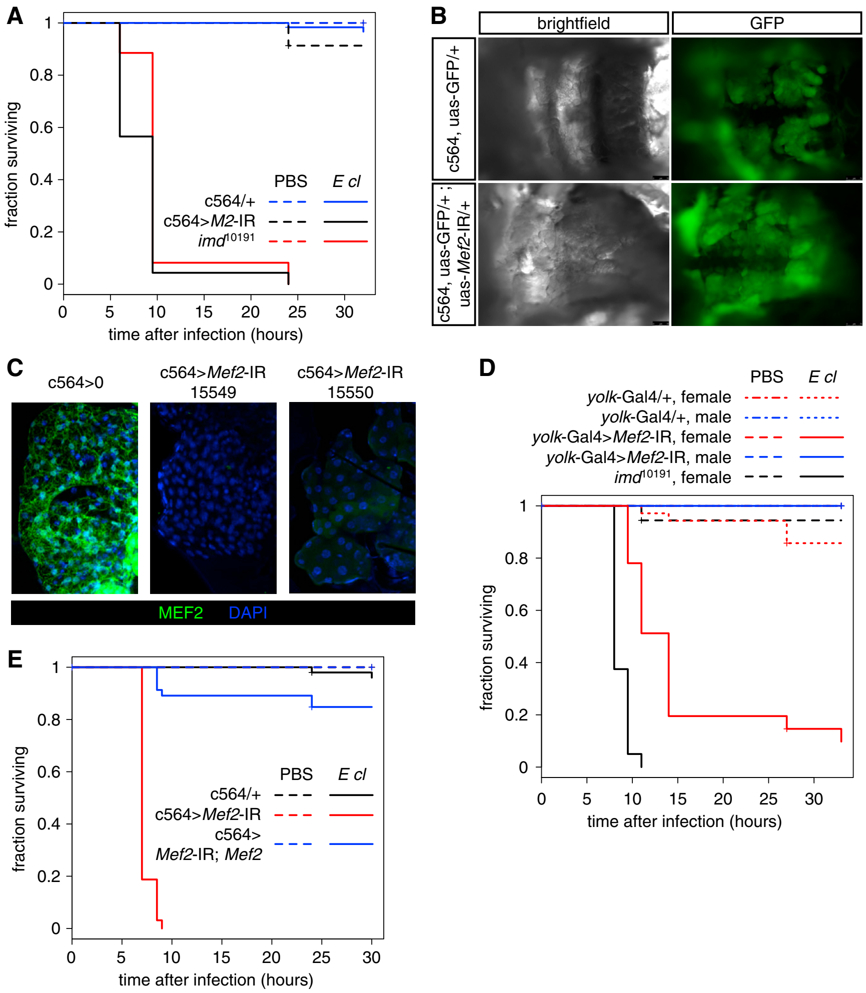

Figure S2. *Mef2* Knockdown Controls and Further Susceptibility Data, Related to Figure 2

(A) Survival of *Mef2* knockdown and *imd* mutant flies is similar after infection with *Enterobacter cloacae*. P values for differences in survival time in infected animals by log-rank test: *Mef2-IR* versus control, p = 0; *Mef2-IR* versus *imd*, p = 0.00062; *imd* versus control, p = 0.

(B) The fat body is present in flies with *Mef2* knocked down under control of c564. Male flies were immobilized, the ventral cuticle was removed from the abdomen, and then the gut, gonad and malphigian tubules were removed, revealing the fat body associated with the dorsal abdominal cuticle (labeled here with GFP driven by c564). No consistent differences were visible in cell or organ size or morphology.

(C) MEF2 protein is reduced in fat body from flies with *Mef2* knocked down under control of c564. Dissected fat body isolated from driver-only controls or *Mef2* knockdowns was immunostained with rabbit anti-MEF2 (green) and nuclei were counterstained with DAPI (blue). Single confocal sections are shown.

(D) *yolk*-Gal4 driven *Mef2* knockdown also gives susceptibility to *E cloacae* infection. Male flies carrying *yolk>Mef2-IR* do not show a susceptibility phenotype because expression of *yolk*-Gal4 is female-specific (Georgel et al., 2001). P values for differences in survival time in infected animals by log-rank test: female *yolk>Mef2-IR* versus female control, p = $2.64{*}10^{-12}$; male *yolk>Mef2-IR* versus male control, p = undefined (no deaths in either genotype).

(E) Susceptibility to *E cloacae* infection in *Mef2*-knockdown flies can be rescued by coexpression of *Mef2*. P values for differences in survival time in infected animals by log-rank test: *Mef2-IR* versus control, p = 0; *Mef2-IR* versus *Mef2-IR*; *Mef2*, p = 0; control versus *Mef2-IR*; *Mef2*, p = 0.06.

Cell

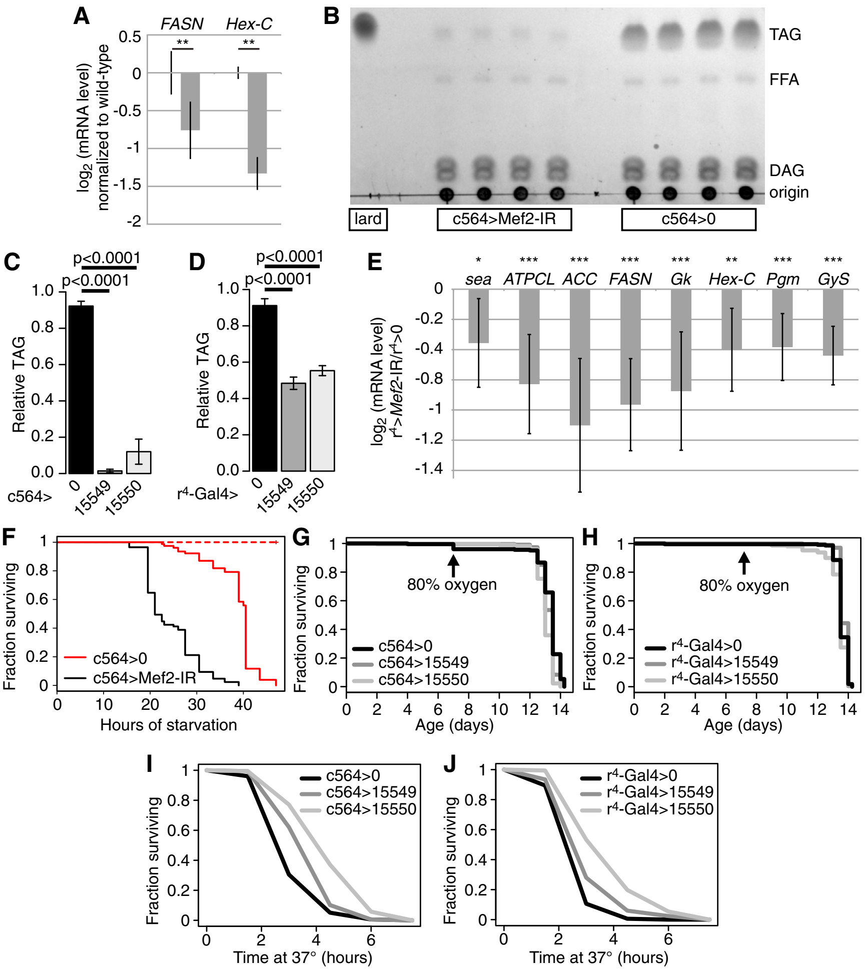

Figure S3. Metabolism and Stress Resistance in *Mef2-IR* Flies and Controls, Related to Figure 3

(A) Expression of *FASN* and *Hex-C* are impaired by expression of a *Mef2-engrailed* repressor fusion protein. Mef2-*EnR*-expressing (*w,* UAS-*Mef2-EnR* / Y ; tub-*Gal80*^{ts} / + ; tub-*Gal4* / +) and control (*w,* UAS-*Mef2-EnR* / Y ; + ; +) flies were maintained at 18° until three days before sample collection, when they were shifted to 29°. mRNA levels are normalized to the control genotype and expressed as $\log_2$. Values are mean ± standard deviation. Significance levels: *$p < 0.01$; **$p < 0.001$, by heteroscedastic unpaired two-tailed t test.

(B) Example thin-layer chromatographic analysis of triacylglycerols (TAG) and other neutral lipids (free fatty acids, FFA; diacylglycerols, DAG) in lard standard, whole adult male *Mef2*-knockdown flies, and whole adult male driver-only controls.

(C) Quantification of TLC data for triacylglycerol in flies with c564-driven *Mef2* knockdown. Levels are normalized to a lard control.

(D) Quantification of TLC data for triacylglycerol in flies with r^4-driven *Mef2* knockdown. Levels are normalized to a lard control.

(E) Expression of metabolic *Mef2* targets in flies with r^4-driven *Mef2* knockdown. Assayed by qRT-PCR. mRNA levels are normalized to the control genotype and expressed as $\log_2$. Values are mean ± standard deviation. Significance levels for difference between knockdown (w^{1118} / Y ; ; r^4-*Gal4* / UAS-*Mef2*-IR) and wild-type control (w^{1118} / Y ; ; r^4-*Gal4* / +): *$p < 0.05$; **$p < 0.01$; ***$p < 0.001$ by heteroscedastic unpaired two-tailed t test.

(F) c564 > *Mef-IR* flies are sensitive to starvation. Flies were starved on 0.02x PBS in 1% agar until dead. Survival of *Mef2* knockdowns is significantly shorter than controls (p = 0 by log rank test).

(G and H) c564 or r^4-driven *Mef2* knockdown does not strongly change time to death due to hyperoxia.

(I and J) c564 or r^4-driven *Mef2* knockdown does not strongly change time to death due to heat stress (37°).

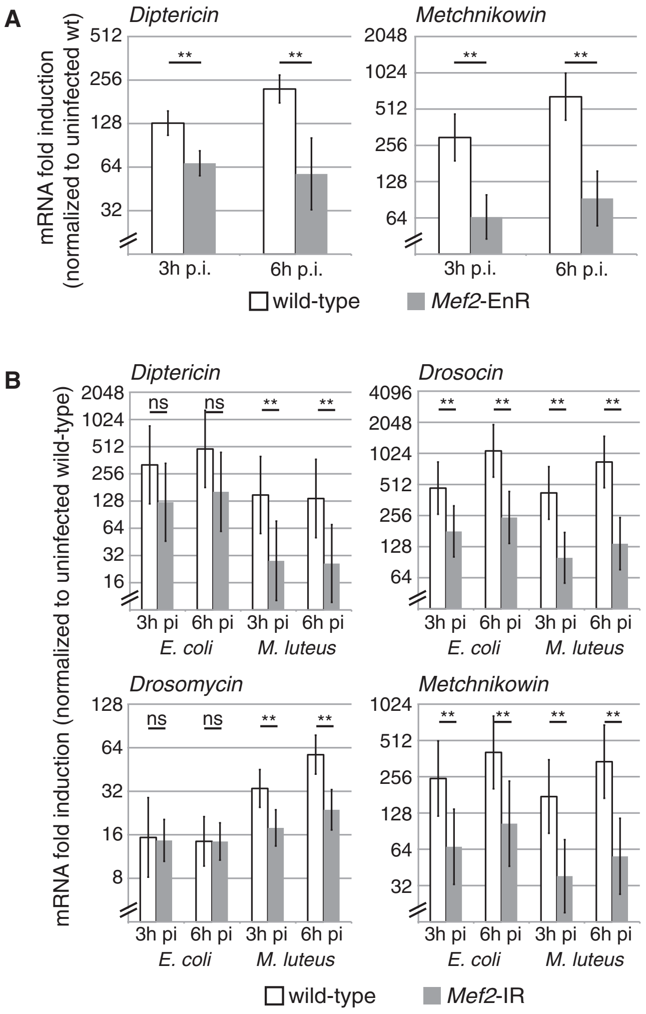

Figure S4. Antimicrobial Peptide Expression in Flies Expressing *Mef2*-EnR or Receiving Separate *M. luteus* and *E. coli* Infections, Related to Figure 4

(A) Induction of *Diptericin* and *Metchnikowin* are impaired by expression of a *Mef2-engrailed* repressor fusion protein. Mef2-*EnR*-expressing (*w,* UAS-*Mef2-EnR* / Y ; tub-*Gal80*^{ts} / + ; tub-*Gal4* / +) and control (*w,* UAS-*Mef2-EnR* / Y ; + ; +) flies were maintained at 18° until three days before infection, when they were shifted to 29°. Samples collected 3 and 6 hr after infection (p.i.).Values are mean ± standard deviation. Significance levels: *p < 0.01; **p < 0.001, by heteroscedastic unpaired two-tailed t test.

(B) Expression of four antimicrobial peptides in *Mef2*-knockdown and control flies after infection with *E. coli* (Gram-negative, imd agonist) or *M luteus* (Gram-positive, Toll agonist), as assayed by qRT-PCR, normalized to uninjected controls. Samples collected 3 and 6 hr after infection (p.i.). Values are mean ± standard deviation. Significance levels: *p < 0.01; **p < 0.001, by heteroscedastic unpaired two-tailed t test.

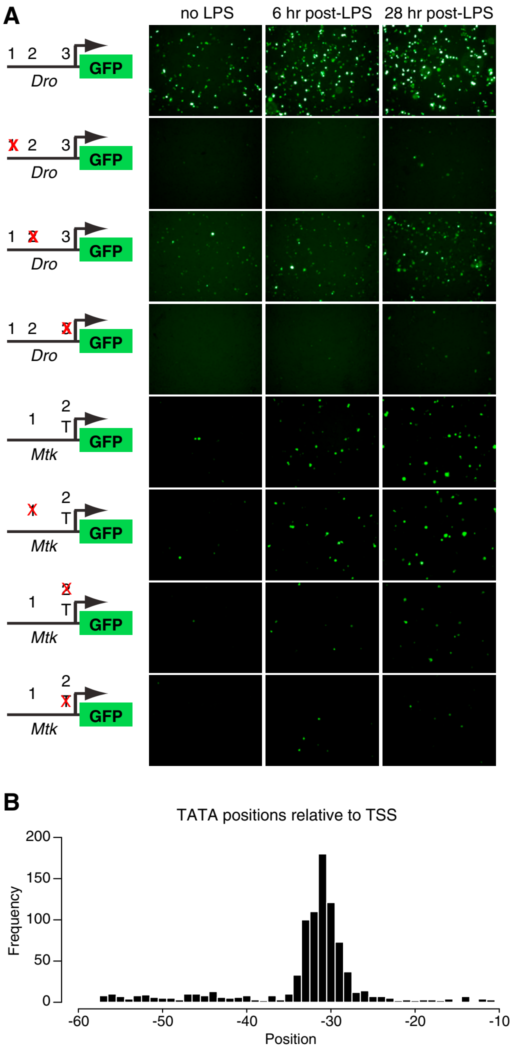

Figure S5. GFP Expression by Antimicrobial Peptide Promoter Constructs, Related to Figure 5

(A) Basal or LPS-induced eGFP expression in S2* cells transfected with constructs as shown in Figure 5. White spots indicate saturated fluorescence signal.

(B) Positions of TATA boxes relative to the transcription start site in the unbiased set.

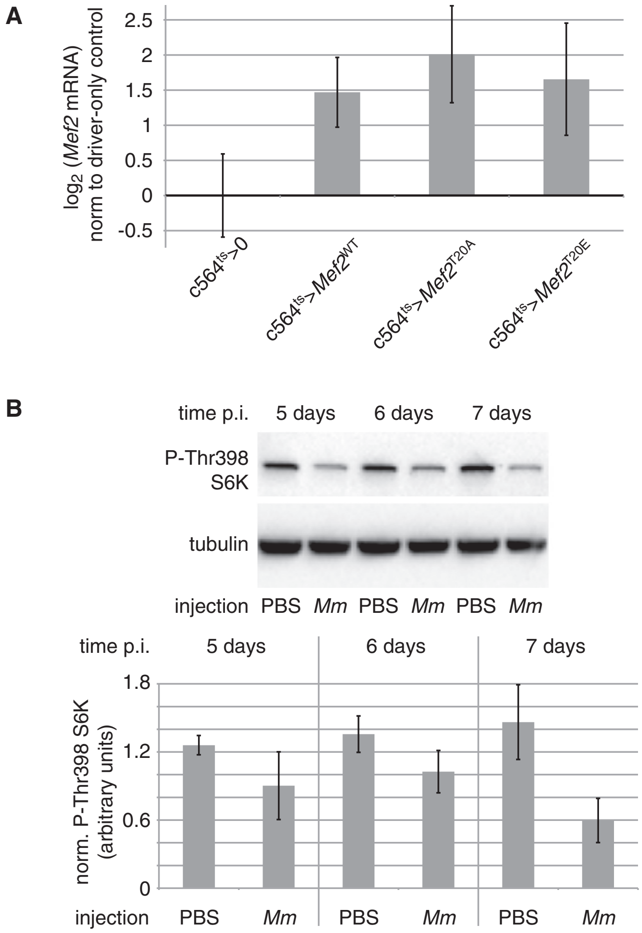

Figure S6. *Mef2* Mutant Misexpression and S6K Regulation by Mycobacterial Infection, Related to Figure 6

(A) Expression of *Mef2* mRNA in driver-only controls and in *Mef2* wild-type and mutant misexpressing lines, assayed by qRT-PCR. Expression shown pools *Mef2* levels from all experimental conditions for each line. Values shown as mean ± standard deviation.

(B) p70 S6K Thr398 phosphorylation is reduced in late stages of *M marinum* infection. Top, representative western on whole-fly lysates, probed with the antibodies indicated; bottom, quantification of phospho-S6K normalized to tubulin from three independent experiments. Infectious dose was 500 CFU; temperature was 25°; all infected flies had died within 24 hr of the 7 day time point. Error bars represent standard deviation.

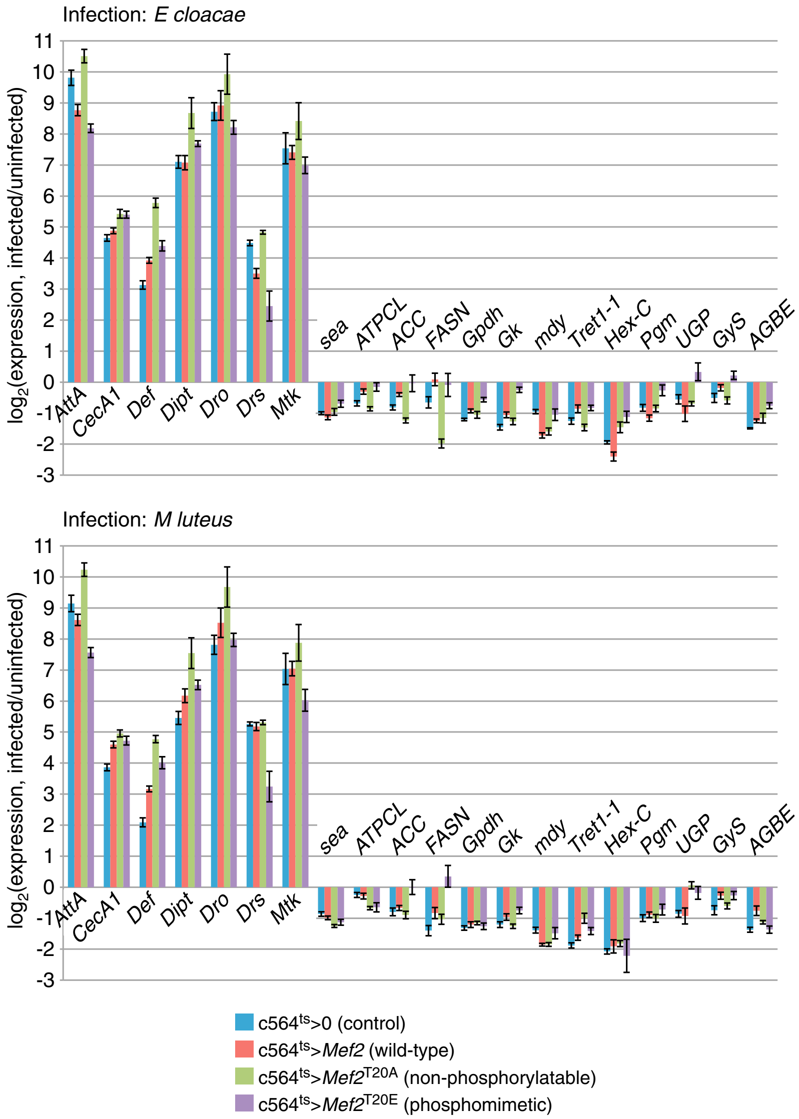

Figure S7. Expression of *Mef2* Targets in Flies Misexpressing *Mef2* Mutants Showing Variability, Related to Figure 7

Inducible fat body expression of wild-type, T20A or T20E *Mef2* using c564; tub-Gal80^{ts}. *Mef2* expression was activated beginning 16 hr before infection. Expression is normalized to the same genotypes, uninjected, collected at the same time after temperature shift. All measurements are the mean of three biological replicates. Values are mean ± SEM.
